# Abstracts from a joint meeting between Medstar Georgetown Diabetic Limb Salvage and the Wound Healing Foundation conference, 10–12^th^ April 2025, Washington DC, USA

**DOI:** 10.1111/iwj.70681

**Published:** 2025-06-02

**Authors:** Laura K. S. Parnell, John Steinberg

**Affiliations:** ^1^ Wound Healing Foundation and Precision Consulting Missouri City Texas; ^2^ MedStar Health Georgetown University School of Medicine Washington DC

The Medstar Georgetown Diabetic Limb Salvage and the Wound Healing Foundation, both nonprofit organizations, recently held their 2025 meeting on the diabetic foot and healing chronic wounds in Washington D.C., USA. These groups chose the *International Wound Journal* to feature their submitted abstracts to highlight the excellent research ongoing in this important clinical area. If you wish to contact any of the authors to discuss their research, please contact us and we can connect you.

The top ten abstract authors, denoted below, were given podium presentations during the meeting in honor of Greg Schultz, PhD. At the time of his passing, Dr. Schultz was the Award Chair and a Board of Director of the Wound Healing Foundation and a Course Director for the joint meeting. Dr. Schultz was a consistent champion for quality clinical and bench science focused on improving wound healing. Presenting the best science in Dr Schultz's honor at the conference seemed like a natural extension to his legacy. Monitor https://www.woundhealingfoundation.org/g‐schultz‐in‐memoriam‐2/ for updated information on a future additional program honoring Professor Shultz in accordance with his family's wishes.

Editorial Note: We recognize the valued contribution of Professor Greg Schultz and his connection with the IWJ. We are pleased to be able to continue honoring his legacy in wound healing.

## 1 TOP 10 ABSTRACTS RECOGNIZED IN HONOR OF GREGORY SCHULTZ, PHD

## 2 101

### 2.1 DNA Methylation Signatures as Predictive Biomarkers for Recurrence and Prognosis in Diabetic Foot Ulcers

#### 2.1.1 Seung‐Kyu Han, MD, PhD; Gordon Lee, MD, FACS; Sik Namgoong, MD, PhD

2.1.1.1

**Background:** Diabetic foot ulcers (DFUs) exhibit poor healing, yet the underlying molecular mechanisms remain unclear. DNA methylation plays a critical role in cellular processes such as proliferation and differentiation, potentially influencing disease progression. We hypothesized that distinct DNA methylation patterns could serve as prognostic biomarkers and therapeutic targets for DFUs.

**Methods:** We analyzed 48 blood samples from 36 DFU patients treated at Korea University Guro Hospital between October 2019 and November 2021. Genome‐wide DNA methylation profiling was performed using the Illumina MethylationEPIC (850k) array to identify differentially methylated regions (DMRs) between patients with good and poor prognoses. Heatmaps were generated to visualize DMRs, and pathway enrichment analyses (KEGG, GO, gene‐concept network, and GSEA) were conducted. A decision tree model was applied to identify key classifiers associated with recurrence.

**Results:** A total of 92 hypermethylated and 108 hypomethylated DMRs (|Log2 fold change| > 0.1, *p* < 0.03) were identified. Among patients with good prognoses, 69 hypermethylated and 156 hypomethylated DMRs were detected. KEGG analysis highlighted the MAPK signaling pathway as a dominant feature. The decision tree model identified MORN1 hypomethylation and NCOR2 hypermethylation as key predictors of recurrence.

**Conclusions:** MORN1 and NCOR2 methylation patterns may serve as potential biomarkers for predicting DFU recurrence and prognosis. DNA methylation profiling offers valuable insights into the molecular mechanisms driving inflammation and poor wound healing in DFUs, paving the way for targeted therapeutic strategies.

## 3 102

### 3.1 Psychological Distress and Calcanectomy History Are Key Predictors of Low Function in Lower Extremity Free Tissue Transfer Patients

#### 3.1.1 Christopher Attinger, MD; Sami Ferdousian, MS; Ryan Lin, MD; Rachel Rohrich; Isabel Snee

3.1.1.1

**Background:** Functional outcomes in microsurgical free tissue transfer (FTT) patients vary and there is a lack of quality of life data. This study uses patient‐reported outcome measures (PROMs) to identify patient factors associated with low functional status after lower extremity (LE) FTT.

**Methods:** Using a cross‐sectional study design, we reviewed patients who underwent LE FTT and answered postoperative surveys from June 2022 to May 2024. PROMs assessed function (LEFS), pain (PROMIS‐3a), psychological distress (SRQ‐20), and resilience (CD‐RISC). Responses were classified as low function (LEFS < 40) or normal function (LEFS =40).

**Results:** Of 114 survey sets, 49 were low function, and 40 were normal function. The mean Charlson Comorbidity index was 4.0 ± 2.1. The low function group had a significantly higher BMI (30.4, IQR: 6.9 vs. 33.3, IQR: 11.0; *p* = 0.018), and proportion of males (30 [61.2%] vs. 4 [10.3%]; *p* = 0.003). The low function group demonstrated a higher prevalence of psychiatric diagnosis (11 [22.5%] vs. 2 [5.1%], *p* = 0.033) and history of calcanectomy (23 [46.9%] vs 9 [22.5%], *p* = 0.026). The normal function group reported lower pain intensity (4, IQR: 3 vs. 7, IQR: 7, *p* = 0.003) and less psychological distress (1, IQR: 3 vs. 4.5, IQR:5, *p* < 0.001). Adjusting for significant covariates, multivariate regression demonstrated that a history of calcanectomy and higher psychological distress were independently associated with low functional status.

**Conclusions:** Addressing factors associated with low function with a multidisciplinary team approach may optimize function. More rigorous preoperative functional assessments are needed to understand function in this complex population.

## 4 103

### 4.1 Dedicated Limb Hospitalist Improves Perioperative Health Outcomes in Patients Undergoing Lower Extremity Reconstruction Treatment for Atraumatic Wounds: Preliminary Results from A Historical Control Study

#### 4.1.1 Christopher Attinger, MD; Sami Ferdousian, MS; Winni Li; Christopher Ply; Rachel Rohrich

4.1.1.1

**Background:** Patients undergoing complex lower extremity procedures for atraumatic wound treatment, such as free tissue transfer (FTT) or below knee amputation (BKA), often present with significant comorbidities that require intensive in‐hospital management. To address these complex needs, a dedicated limb hospitalist was integrated into the care team at MedStar Georgetown University Hospital (MGUH). In June 2024, however, the limb hospitalist role became vacant. We hypothesized that the presence of a dedicated limb hospitalist would be associated with improved clinical outcomes in this medically complex population.

**Methods:** We conducted a historical control study comparing patients who underwent either an FTT or BKA at MGUH from two periods: (1) while a limb hospitalist was on staff (January 2024 to June 2024; n=53), and (2) after the hospitalist left (June 2024 to January 2025; n=57). Primary outcomes included length of stay (LOS), postoperative LOS, complications requiring ICU management, and 30‐day hospital readmission rates.

**Results:** A total of 110 patients were evaluated (48.2% with a dedicated limb hospitalist; 51.8% without). The overall median LOS was 22 days in both groups (p = 0.939). Of note, in patients undergoing BKA, the median postoperative LOS was significantly shorter when managed by the dedicated limb hospitalist (9.5 vs 13 days, *p* = 0.018). For patients discharged to an inpatient rehabilitation facility, the LOS at the facility was also lower in the hospitalist group (15.6 vs 17.0 days, *p* = 0.586). While not statistically significant, the group with the dedicated limb hospitalist also demonstrated lower rates of complications requiring ICU management (9.4% vs 15.8%, *p* = 0.397) and fewer 30‐day readmissions (7.6% vs 12.3%, *p* = 0.530). Additionally, trends toward reduced rates of inpatient‐developed thrombolytic complications (1.9% vs. 5.3%; *p* = 0.619), adverse cardiac events (3.8% vs. 8.8%; *p* = 0.440), and sepsis (7.6% vs. 10.5%; *p* = 0.744) were noted in the hospitalist cohort, although these did not reach statistical significance.

**Conclusions:** In this small cohort, having a dedicated limb hospitalist on staff was associated with a significant reduction in postoperative LOS, as well as clinically meaningful trends toward fewer ICU‐level complications and 30‐day hospital readmissions. Although many differences did not reach statistical significance (likely reflecting the modest sample size), these findings highlight the potential value of a dedicated hospitalist in optimizing care coordination and medical management for complex limb salvage patients, also while potentially lowering healthcare utilization and costs (ICU and readmission). Sustaining a dedicated limb hospitalist position could therefore be an important investment in improving quality of care in this high‐acuity surgical population.

## 5 104

### 5.1 The Benefits of Ongoing Treatment with High‐Concentration Capsaicin Topical System (HCCTS) on Affective Distress and Quality of Life (QoL) in Patients with Painful Diabetic Peripheral Neuropathy (PDPN) of the Feet: The CASPAR Real‐World Study

#### 5.1.1 Sam Allen PhD; Marielle Eerdekens, MD, MBA; Sunrut Patel, MD; Tamara Quandel, PhD; Michael Überall, MD, PhD

5.1.1.1

**Background:** Chronic PDPN develops in 30% to 50% of people with DPN.1‐3 PDPN of the feet contributes to an estimated 6.8 million lower‐limb amputations worldwide annually,4 and the pain can be particularly debilitating, interfering with daily activities, causing disability and psychosocial impairment, and affecting sleep, ability to work, mood, and QoL.5 Changes in pain intensity following one to four HCCTS treatments along with shifts in affective distress, QoL, and sleep were evaluated over 12 months in a real‐world patient population.

**Methods:** CASPAR, a noninterventional, retrospective cohort study, included patients with PDPN of the feet who received =1 HCCTS treatment (~3‐month intervals) and were followed for 12 months. Data from the German Pain eRegistry were used to assess pain intensity (VAS) and QoL via the Depression, Anxiety, and Stress Scale (DASS‐21), Veterans RAND‐12, and modified pain disability index (mPDI) sleep item and suicidal ideation.

**Results:** Significant improvements were observed for all patients (n=365) who received one to four HCCTS treatments, with the most significant changes observed in those who received the maximum four treatments (Figures 1A‐G). With discontinuation of HCCTS, scores worsened and/or reverted to baseline. By the fourth treatment, 100% of patients (n=108) achieved =50% reduction in pain. Concomitant pain medication use decreased with continued treatment, and 34% of patients receiving four HCCTS applications were able to discontinue all concomitant analgesic medications at 12 months (Figure 1H).

**Conclusions:** Ongoing HCCTS treatment was well tolerated and associated with progressive improvement across assessments of pain, affective distress, sleep, suicidal ideation, and QoL.

## 6 105

### 6.1 The Impact of Nitric Oxide‐Generating Wound Dressing in Diabetic Foot Ulcers in Patients Receiving Antibiotics: Post‐Hoc Analysis

#### 6.1.1 Michael Edmonds, MD; Alan Horner, PhD; Chris Manu, MD; Daniel Metcalf, PhD

6.1.1.1

**Background:** Diabetic foot ulcers (DFUs) result in poor patient quality of life and substantial economic burden. Nitric oxide (NO) within a wound dressing represents a promising agent for use in DFU treatments due to its antimicrobial properties. The purpose of this analysis was to evaluate the impact of a prototype NO‐generating dressing (NOGD), compared with standard of care (SoC), on DFU healing in patients receiving antibiotics.

**Methods:** A post‐hoc analysis of the ProNOx1 randomized controlled trial (Edmonds et al., 2018 [1]) was performed to determine the impact of NOGD on DFU healing in patients receiving antibiotics at commencement and/or during the trial. Primary efficacy measure of this post‐hoc analysis was DFU percent area reduction (PAR) at 12 weeks. Secondary efficacy measure was number of DFUs healed at 12 weeks.

**Results:** Of the 124 patients that were treated per protocol, 63 (33 in the SoC population; 30 in the NOGD population) were treated with antibiotics. At 12 weeks, mean PAR was 13.6% and median PAR was 44.3% in the SoC population, compared to mean PAR of 61.5% and median PAR of 87.1% in the NOGD population. At 12 weeks, the number of healed DFUs was 5/33 (15%) in the SoC population and 11/30 (37%) in the NOGD population.

**Conclusions:** This post‐hoc analysis of DFUs in patients receiving antibiotics, which can be assumed to be infected or at‐risk of infection, demonstrates the ability of NOGD to improve healing outcomes compared with SoC.

## 7 106

### 7.1 A National Trend Analysis of Primary Targeted Muscle Reinnervation with Below‐Knee Amputation

#### 7.1.1 Sami Ferdousian, MS; Grant Kleiber, MD; Christian Lava, MS; Ryan Lin, MD; Rachel Rohrich

7.1.1.1

**Background:** Residual and phantom limb pain are common following below‐knee amputation (BKA). The use of concurrent targeted muscle reinnervation (TMR) at the time of BKA has recently been introduced to prevent neuropathic pain and enhance prosthetic control. This study aims to evaluate the national trends of primary TMR with BKA.

**Methods:** Encounters of BKA with and without TMR from 2020 and 2021 were identified using ICD‐10 procedural codes in the National Inpatient Sample (NIS) database. NIS weights were applied to generate national estimates. Diagnostic codes were used to identify patient comorbidities during hospital admission. Between group analysis was conducted to identify patient‐ and hospital‐related characteristics associated with immediate TMR.

**Results:** A total of 95,395 weighted BKA encounters were identified, of which 760 (0.8%) received immediate TMR. There was a 66.7% relative increase in the incidence of immediate TMR from 2020 to 2021. Overall, TMR was associated with a younger age 55.3 ± 1.1 vs. 61.3 ± 0.1, *p* < 0.001) and higher median residential income (p < 0.001). Average hospital length of stay (13.4 ± 0.1 vs. 13.1 ± 1.5, *p* = 0.828) and total hospital costs (172,231 ± 2,581.5 vs. 191,779.6 ± 21,747.1 dollars, *p* = 0.366) were similar between TMR and Non‐TMR groups. Medicare was the most common payer in both groups (TMR: 39.5% vs. Non‐TMR: 55.5%), but TMR demonstrated a significantly higher incidence of private insurance use (31.6% vs. 17.7%, *p* = 0.0011). Those receiving immediate TMR had significantly lower rates of end‐stage renal disease (10.5% vs. 18.5%, *p* = 0.0109), chronic kidney disease (30.3% vs. 45.0%, *p* = 0.0029), diabetes mellitus type‐2 (50.0% vs. 75.9%, *p* < 0.001), and peripheral vascular disease (18.4% vs. 29.9%, *p* = 0.0029). Patient characteristics associated with TMR remained consistent from 2020 to 2021. TMR procedures were significantly more likely to be conducted in an urban location (99.3% vs. 92.5%, *p* = 0.0015) and in the Midwest (34.9% vs. 21.4%, *p* = 0.0140) compared to Non‐TMR BKA. Multiple variable regression identified higher residential income, DM‐2, PVD, treatment in a private investor‐owned hospital, urban teaching hospitals, and hospital location in the Midwest as independent predictors of TMR utilization.

**Conclusions:** Despite growing clinical evidence demonstrating the efficacy of TMR in reducing postoperative pain among BKA patients, it is performed in less than 1% of cases. Access to immediate TMR with BKA varies significantly based on patient demographics, comorbid conditions, and hospital location. To enhance postoperative outcomes and quality of life for patients undergoing BKA, efforts should be made to implement TMR in broader geographical and patient contexts.

## 8 107

### 8.1 Correlation Between Objective and Subjective Measures to Achieve Functional Limb Salvage

#### 8.1.1 Olivia B. Allen, BA; Ann Hu, MS1; Meghan E. Currin, BA; Kishan S. Shah , BS; Jie Shih BA, MS; Luke J. Llaurado, BA; Holly D. Shan, BS; Rachel N. Rohrich, BS; John S. Steinberg, DPM; Richard C. Youn, MD; Christopher Attinger, MD; Karen K. Evans, MD; Jayson N. Atves, DPM

8.1.1.1

**Background:** Functional ambulation is a key component of limb salvage in the chronic lower extremity (LE) wound population. Function can be measured subjectively using patient reported outcome measures, specifically the lower extremity functional scale (LEFS) and objectively using wearable gait sensors. This study evaluates the relationship between gait parameters and PROMs to identify patients at risk for adverse outcomes.

**Methods:** Between December 2021 and February 2024, adult patients who could ambulate unassisted, without pain, who had no open wounds, and had not undergone lower extremity surgery in the preceding three months, were eligible for participation. Participants completed a standardized assessment protocol using wearable gait sensors, completing a 120‐second walk test at a self‐selected pace. LEFS surveys were given to patients at the beginning of the visit and were then stratified to Moderate‐High (MH) function (scores 41‐80) and Mild‐Minimal (MM) function (0‐40). The gait sensor data was analyzed using the Motility Lab software and STATA VSN 8.0 with statistical significance set at *p* < 0.05.

**Results:** A total of 104 patients were included for analysis. For the MM group the average age was was 56.0, and average BMI was 32.6. In the MH group, the average age was 58.7, and BMI was 29.8. There was a higher proportion of females in the Mild‐Minimal function group compared to the Moderate‐High (70.1% vs. 26.3%, *p* < 0.05 (Table 1). There were no differences between the MM vs MH groups in proportion of smokers (29.2% vs. 26.3%, *p* = 0.796), peripheral neuropathy (33.3% vs. 26.3%, *p* = 0.604), Diabetes Mellitus Type II (33.3% vs. 32.5%, *p* =1.000), chronic kidney disease (20.0% vs. 8.8%, *p* = 0.273), history of a minor lower extremity amputation (LEA) (28.6% vs. 71.4%), or history of a major LEA (45.5% vs. 54.6%). Patients with MM function had slower gait speed (0.64 ± 0.05 vs. 0.95 ± 0.01, *p* < 0.001), longer step duration (0.67 ± 0.03 vs. 0.58 ± 0.01, *p* < 0.001), decreased cadence (78.51 ± 3.60 vs. 103.39 ± 1.31, *p* < 0.001), decreased single limb support (33.06 ± 0.73 vs. 37.45 ± 0.25, *p* < 0.001), increased double limb support (25.82 ± 2.32 vs. 24.51 ± 0.49, *p* < 0.001), decreased stride length (0.61 ± 0.40 vs 0.83 ± 0.01, *p* < 0.001) (Table 2).

**Conclusions:** Patients with decreased LEFS scores demonstrated pathologic gait even in the setting of comparable comorbidities. This study has correlated objective analysis using gait sensors with PROMs to understand and correlate ambulatory status. Plastic surgeons should consider PROMs for screening in high‐risk wound care patients.

## 9 108

### 9.1 See Your Feet: A Pilot Study Evaluating a Telescopic Mirror Device for Diabetic Foot Ulcer Prevention

#### 9.1.1 Christopher Attinger, MD; Karen Evans, MD; Rachel Rohrich; Ella Song; Richard Youn, MD

9.1.1.1

**Background:** Diabetic foot ulcers (DFUs) are a common yet serious complication of diabetes, affecting approximately 15% of diabetic patients. If left untreated, DFUs pose major risk as they are currently the leading cause of non‐traumatic lower‐limb amputation, which impacts mobility and quality of life. In patients with peripheral neuropathy, DFUs often develop insidiously due to diminished protective sensation, and are often identified only after significant tissue loss. Detection of DFUs are further complicated in obese patients with limited mobility who may struggle to visually inspect the plantar surface of their feet. In order to address these challenges, we developed a device to allow users to easily view hard‐to‐see areas of their feet. In this pilot study, we aim to evaluate the device's ease of use, utility, and effectiveness among diabetic patients.

**Methods:** A “See Your Feet” device was designed using a telescopic mirror with an LED light attached to a wand and distributed to 10 patients with diabetes. A mixed‐methods qualitative and Likert‐scaled survey was conducted with 10 diabetic patients who utilized the device over a two‐week period to inspect their feet. Questions evaluated the device's usability and effectiveness. Patients were also asked to state their willingness to continue using the device post‐trial.

**Results:** All participants (n=10) completed the survey. Responses indicated high usability, with all users reporting that the device was “easy” or “very easy” to use. The device was rated as “effective” or “very effective” in helping users clearly view their feet. Most participants (70%) stated they would continue using the device after the trial, citing improved confidence in their ability to detect potential foot‐related issues. Additionally, 20% of participants noted visualizing foot‐related issues during the trial period that they believed warranted communication with their healthcare provider.

**Conclusions:** This pilot study demonstrates the potential of the “See Your Feet” telescopic mirror device to improve foot self‐monitoring among diabetic patients, particularly those with mobility limitations. The device was well‐received and deemed effective for routine foot inspections among all participants. These findings support the need for a larger‐scale study to further validate these observed benefits, especially in underserved populations with limited access to multidisciplinary wound centers. By empowering patients with tools for early detection, the evaluated novel device has the potential to significantly reduce the incidence of DFUs and associated complications.

## 10 109

### 10.1 Peak Plantar Pressure Assessment in the Midfoot: Influence of Diabetic Peripheral Neuropathy

#### 10.1.1 Georgeanne Botek, DPM; Brian Davis, PhD; Ahmet Erdemir, PhD; Jessi Martin‐Liddy

10.1.1.1

**Background:** Charcot Neuropathic Sarco‐Osteoarthropathy (CNSOA) is a degenerative disorder arising from diabetic peripheral neuropathy (DPN), with a 35% risk of development in individuals with DPN. Early diagnosis is crucial for effective treatment and preventing amputation, but misdiagnosis is common due to nonspecific symptoms. Literature shows that early diagnosis (stage 0) significantly improves healing outcomes, while delays increase the risk of foot deformity. However, the effect of DPN severity on foot loading patterns and midfoot pressure changes remains underexplored.

**Methods:** Forty‐two participants (ages 45‐84) were divided into diabetic non‐neuropathic and diabetic neuropathic groups, with neuropathy severity assessed using the Michigan Neuropathy Screening Instrument (MNSI). Peak plantar pressure data were collected with the Novel Pedar‐x insole system and analyzed using MATLAB. The relationship between MNSI scores and peak midfoot pressure was assessed using Pearson's correlation.

**Results:** A statistically significant positive correlation was found between increasing MNSI scores and increased peak midfoot pressure (p = 0.045), indicating that increasing neuropathy severity correlates with greater pressure in the midfoot.

**Conclusions:** Our study demonstrates that as DPN severity increases, so does the peak pressure in the midfoot, reflecting potential early signs of arch collapse. The MNSI test offers a simple, non‐invasive tool to monitor DPN progression and midfoot stability in diabetic patients, supporting early intervention to prevent deformities.

## 11 110

### 11.1 The Ertl Below‐Knee Amputation Technique Improves Gait Function in Nontraumatic Amputees

#### 11.1.1 Rajan M. Negassa, BS; Ademide O. Young, BS; Nori C. Pham, BS; Sirri N. Akaya, BS; Rachel N. Rohrich, BS; Michael Kishek, BS; John S. Steinberg DPM; Richard C. Youn, MD; Jayson N. Atves, DPM; Christopher E. Attinger, MD

11.1.1.1

**Background:** Understanding gait in patients with below‐knee amputations (BKA) is important for evaluating mobility. The Ertl procedure, which creates a tibiofibular bone bridge, theoretically enhances end‐bearing capabilities and increases prosthetic control by recreating the ankle joint. However, its impact on gait parameters has not been explored in the nontraumatic amputee population. Thus, this study evaluates whether the Ertl technique confers functional advantages when compared to the standard Burgess BKA technique.

**Methods:** Between June 2021 and September 2024, adults who could safely ambulate without pain, open wounds, and surgery in the previous three months completed a standardized 120‐second walk test with wearable sensors. Patients who underwent BKA without Ertl (non‐Ertl) were compared to patients who underwent Ertl.

**Results:** A total of 47 patients with BKA were identified, of which 11 were Ertl and 36 were non‐Ertl. Demographic characteristics and comorbidities were similar between groups (*p* > 0.05). Ertl patients had faster gait speed (0.9 ± 0.3 m/s vs. 0.7 ± 0.2 m/s, *p* = 0.028), shorter step duration (0.6 ± 0.1s vs. 0.7 ± 0.1s, *p* = 0.024), and increased cadence (101.1 ± 13.8 steps/min vs. 89.9 ± 11.6 steps/min, *p* = 0.016). No significant differences were observed in elevation at mid‐swing, single and double limb support, and sway.

**Conclusions:** These findings support the theoretical functional advantages of the Ertl procedure over the traditional Burgess technique in the nontraumatic amputee population.

## 12 ABSTRACT POSTERS

## 13 Poster numbers are identified in the order they were submitted

## 14 111

### 14.1 Immediate Recoil After Percutaneous Balloon Angioplasty of Below The Knee Arteries in Patients With Critical Limb Ischemia

#### 14.1.1 Farruk Malik, MD, FACC

14.1.1.1

**Background:** Chronic limb ischemia (CLI), the terminal manifestation of peripheral artery disease, has an estimated prevalence of 6.5 million in the United States and Japan, with an annual incidence of 2.3% per year. Patients diagnosed with CLI have a 1‐year mortality rate of approximately 20% to 25%, and 5‐year all‐cause mortality rates exceeding 50%.Percutaneous transluminal angioplasty [PTA]) of below the knee arteries in critical limb ischemia (CLI) is a common, established practice worldwide. Recoil and restenosis remain major drawback of balloon angioplasty . Durable below the knee vessel patency may promote faster, more sustained, complete wound healing and limb preservation, and is a desired revascularization goal. Plain balloon (PB) angioplasty for below the knee artery disease has a high rate of procedural success and an acceptable safety profile, however, rates of recoil and restenosis are considerable. Balloon dilatation of the diseased artery causes fracture and separation of the media from the intima and stretching of the media and adventitia. Severely fibrotic lesions or heavily calcified lesions are more resistant to balloon dilation, and intimal dissection or elastic recoil may be observed

**Methods:** Our hypothesis was that Immediate recoil, defined as lumen compromise > 10%, is frequent and accounts for considerable luminal narrowing after below the knee angioplasty, promoting restenosis. To test this theory, 25 consecutive CLI patients (17 men and 8 women; mean age 69.2 ± 12.1 years) were angiographically evaluated immediately after balloon angioplasty and 15 min later. 96% of the patients were diabetics. Target lesions included anterior and posterior tibial arteries and the peroneal artery with/without the tibioperoneal trunk. Early elastic recoil was determined on the basis of minimal lumen diameter (MLD) measurements at baseline (MLDbaseline), immediately after balloon angioplasty (MLDpostdilation), and 15 min thereafter (MLD15min).Percentage of the recoil was determined by comparing Minimal Luminal Diameter (MLD) Post angioplasty and then MLD at 15 min using Fluoroscopic Markers in Catheterization Lab. The degree of recoil was correlated with six variables: patient age and sex, comorbidities including diabetes, lesion location, lesion length, and length of the peripheral balloon used.

**Results:** Immediate Elastic recoil was observed in 19 (79%) patients with a mean luminal compromise of 31% according to MLD measurements (MLDbaseline 0.25 mm, MLD postdilation 2.0 mm, MLD immediately 1.55 mm and MLD15min 1.57 mm).

**Conclusions:** Immediate recoil is frequently observed in CLI patients undergoing below knee angioplasty and may significantly contribute to restenosis. These findings support the role of dedicated mechanical scaffolding approaches for the prevention of restenosis in below the knee arteries.

## 15 112

### 15.1 Skin‐Sparing Debridement in NSTI is Possible to Both Limb‐Salvage and Lifesaving ‐4‐Year Single‐Center Retrospective Analysis

#### 15.1.1 Chikashi Morikawa, MD

15.1.1.1

**Background:** Necrotizing soft tissue infections of the lower extremities are highly emergent and fatal, requiring early emergency debridement. It has been reported that 25‐50% of patients require lower extremity amputation to save their lives, while the mortality rate is as high as 16.4‐30.4%. We report here the results of our Skin‐Sparing Debridement, which has been both lifesaving and limb‐salvage.

**Methods:** Patients diagnosed with necrotizing soft tissue infection of the limbs and had Skin‐Sparing Debridement at Yao Tokusyukai General Hospital during 2019‐2022 were revied. Patient factors, causative pathogen and one year mortality are retrieved.

**Results:** 59 patients (11 females and 48 males) were analyzed. Pathogen was identified all patients 52 patients were type 1 polymicrobial . 7 patients were type 2 Streptococcus pyogenes. One year mortality was 86.9%(95% CI 74.2%‐93.6%).Amputation rate was 6.5%(95%CI2.1%‐19%)

**Conclusions:** In all cases, emergency debridement did not require large skin excision(Skin‐Sparing Debridement), and the infection could be controlled. As a result, it was possible to both limb‐salvage and lifesaving. In debridement of necrotizing soft tissue infections, en‐bloc debridement may not always be necessary.

## 16 113

### 16.1 The Efficacy of Powdered Vancomycin in Enhancing Healing Rates and Infection Control of Diabetic Foot Ulcers

#### 16.1.1 Adrienne Gorman, PHD; Camellia Russell

16.1.1.1

**Background:** Diabetic foot ulcers (DFUs) are associated with a significant healthcare burden in the US, forcing out millions of people around the globe. Diabetic foot ulcers are associated with a considerable degree of morbidity and are at high risk for infection, as well as the need for amputation in severe cases. Therefore, improving DFU management is crucial to enhancing patient outcomes and decreasing costs per diabetic‐related complication for the health system. Powered vancomycin has recently received widespread attention as a potentially beneficial adjunctive therapy for DFUs.

**Methods:** We searched PubMed, Embase, Cochrane Library, and Google Scholar systematically using the keywords “powdered vancomycin,” “diabetic foot ulcers,” “healing rates,” and “infection control.” We used this as a search filter by methodological objective (i.e., “powdered vancomycin” Diabetic ulcers in the foot). This review will include studies from January 2000 to present.

**Results:** An analysis of healing rates across the included studies demonstrated an improvement in the group treated with powdered vancomycin. This improvement was statistically significant as compared to control groups. Studies reported an average healing rate of 75% in the powdered vancomycin group compared to the control group (p < 0.05). One study reported a healing rate of 80% at 12 weeks for patients receiving powdered vancomycin [8]. In contrast, other research found a healing rate of 70% at 8 weeks. None of the studies reported any particular side effects from these treatments [5].

Conclusions from these studies support that powdered vancomycin can significantly lower the chance of infections in diabetic foot ulcers. In the powdered vancomycin group, the infection rate fell by 15%. This figure was 30% for the control group (p < 0.01). In particular, it points to a decline in MRSA infections, with just 5% of patients not receiving any treatment developing MRSA complications compared to 20% of the treating group [5].

**Conclusions:** Our systematic review shows some promise for powdered vancomycin in treating diabetic foot ulcers. While some studies indicate improvements in healing time and rate of infection, conflicting data makes it impossible to draw a stable conclusion about vancomycin's overall efficacy. A limitation of this review is that different study designs and background populations of patients make it more difficult to generalize outcomes. Consequently, more research is needed to define the effectiveness and safety of powdered vancomycin in this context. Large, well‐designed, randomized controlled trials are necessary to provide definitive answers and guide clinical practice.

## 17 114

### 17.1 Management of Calcaneal Osteomyelitis

#### 17.1.1 Henna Akbarzai, DPM; Melody John, DPM; Michael Subik, DPM

17.1.1.1

**Background:** The treatment of lower extremity osteomyelitis continues to be a challenge to clinicians. The most definitive treatment for osteomyelitis is resection of infected bone, but this can lead to large bony defects and alterations in biomechanics. This is especially prevalent in the calcaneus which is the major weight bearing tarsal bone. Our case study showcases the treatment of osteomyelitis in the calcaneus using antibiotic coated intraosseous rod by local delivery of antibiotics to the surrounding bone.

**Methods:** A 64 year old female who presented to the hospital for right foot infection after receiving an injection to the right heel for plantar fasciitis. Imaging and pathology revealed acute osteomyelitis of the right talus and calcaneus despite receiving a prolonged course of dual antibiotic therapy. Patient underwent staged surgical approach entailing application of multiplanar external fixator and insertion of antibiotic coated intraosseous rod, and tibiotalocalcaneal arthrodesis.

**Results:** Advanced imaging and pathology revealed acute osteomyelitis of the right talus and calcaneus

**Conclusions:** Calcaneal osteomyelitis is associated with a high degree of disability and risk of hematogenous bacteremia. One surgical treatment includes resection of the infected portions of bone, but this often leads to large bony defects and devastating effects on biomechanics. Many studies have described successful treatment of lower extremity osteomyelitis using the Masquelet technique with antibiotic impregnated cement. Our case study showcases the successful treatment of calcaneal osteomyelitis using an antibiotic coated intraosseous rod through local elution of antibiotics to surrounding bone, thus preserving the limb and its biomechanics.

## 18 115

### 18.1 A Mechanism of Action of an Inflammatory Cytokine Inhibiting Solution

#### 18.1.1 Daniel Gibson, PhD; Laura K. S. Parnell, MS

18.1.1.1

**Background:** Tumor necrosis factor‐a (TNF‐a) is a potent cytokine at the center of many tissue‐destroying immunological processes(1). Acute and chronic wounds with impaired healing and can have excessive inflammation with abnormally high levels of TNF‐a(2). A biologics‐based solutions (Benova, Benova LLC) has been observed to reduce inflammation in ulcers, and an initial laboratory‐based mechanistic study found a decrease in detectable TNF‐a via ELISA(2). Herein experiments were conducted to determine if the mechanism was by hydrolysis of the TNF‐a.

**Methods:** Recombinant TNF‐a (50 μg at 250 ng/ml) was co‐incubated with the test solution or the vehicle and the reaction was periodically sampled. The samples were resolved on a SDS‐PAGE gel and compared to a standard curve of TNF‐a, the test solution, the vehicle, and a molecular weight ladder. The experiment was run in quadruplicate. The gels were stained and scanned for molecular weight determination and relative quantification of the bands. Separate gels were used to generate a 2‐fold dilution standard curve of TNF‐a for quantification via integrated density.

**Results:** During staining, one gel was lost due to a fall. For the molecular weight standard curve, a 3rd order equation was fit (R2 = 0.9998) In the test solution, but not the vehicle, two degradation products were observed at 10.8 kDa and 8.6 kDa. These bands were present at the first, 2 min, time point, and appeared to remain stable through the 30 min time point. A quadratic equation was fit to the TNF‐a standard curve (R2 = 0.9800). The intact TNF‐a doublet was reduced by the action of the active ingredients (range: 9‐19% ; 22 – 49 ng/ml of TNF‐a).

**Conclusions:** The undisclosed active ingredients appeared to hydrolyze the TNF‐a. Using technologies to reduce TNF‐a activity may be helpful in promoting an environment more conducive for healing. Clinical observations of improvements in inflammation and healing to closure following product application suggest a potential correlation with reducing TNF‐a. Additional testing with more sensitive methods would allow testing at physiological concentrations of TNF‐a.

## 19 116

### 19.1 Functionally Enhanced Human Adipose‐Derived Stem Cells Facilitate Wound Healing and Revascularization in Diabetic Foot Ulcer: In Vitro and In Vivo Study

#### 19.1.1 Namkyu Lim, MD, PhD

19.1.1.1

**Background:** Diabetes mellitus accounts for 15%–25% of all chronic foot ulcers. Peripheral vascular disease worsens diabetic foot disease and contributes to the occurrence of ischemic ulcers. Utilizing cell‐based therapies is a viable approach to repair damaged blood vessels and stimulate the formation of new ones. Adipose‐derived stem cells (ADSCs) possess the potential to promote angiogenesis and regeneration due to their strong paracrine effect. Ongoing preclinical studies are investigating alternative methods such as genetic modification or biomaterials to enhance the effectiveness of auto‐transplantation of human ADSCs (hADSCs). Unlike genetic modifications and biomaterials, numerous growth factors have already received approval from regulatory authorities.

**Methods:** This research study explored the positive impact of enhanced human ADSCs (ehADSCs) combined with a mixture of fibroblast growth factor (FGF) in promoting the healing of diabetic foot ulcers.

**Results:** In vitro experiments demonstrated that ehADSCs displayed elongated spindle‐shaped morphology and exhibited significantly increased proliferation. Furthermore, ehADSCs demonstrated improved functionality in terms of tolerating oxidative stress, maintaining stem cell properties, and mobility. In an animal model of diabetes induced by streptozotocin, local transplantation of 1.2 × 10^6 hADSCs, ehADSCs or normal saline was performed. The ehADSCs group exhibited a statistically significant reduction in wound size and increased blood flow compared to both the hADSCs group and the sham control group. Some ADSCs‐transplanted animals displayed the presence of human nucleus antigen (HNA) positive cells, with a relatively higher proportion observed in the ehADSCs group compared to the hADSCs group. However, blood glucose levels did not show any significant differences among the groups.

**Conclusions:** In conclusion, ehADSCs demonstrated superior performance in vitro when compared to conventional hADSCs. Moreover, topical injection of ehADSCs into diabetic foot ulcer improved wound healing, blood flow, and histological markers associated with revascularization.

## 20 117

### 20.1 The Impact of Frailty on Patient Reported Outcomes in the Chronic Lower Extremity Wound Population undergoing Free Tissue Transfer

#### 20.1.1 Joshua Carreras; Sami Ferdousian, MS; Ryan Lin, MD; Rachel Rohrich; Isabel Snee

20.1.1.1

**Background:** The modified 5‐factor frailty index (mFI5) has been used to predict adverse outcomes in limb salvage patients. Our study aims to analyze patient reported Outcomes (PROMs) in low and high frailty patients undergoing lower extremity (LE) free tissue transfer (FTT).

**Methods:** Between June 2022 and September 2024, PROMs were collected via QR code for adult non‐healing LE wound patients who underwent FTT and presented to a tertiary wound center. The Self‐Report Questionnaire (SRQ‐20), PROM Information System Pain Intensity (PROMIS‐3a), Connor‐Davidson Resilience Scale (CD‐RISC), and Lower Extremity Functional Scale (LEFS) were completed at 1, 3, and 6 months and 1, 2, and 5 years postoperatively.

**Results:** Of 54 patients, 28 were classified as high frailty (mFI5 = 2) and 26 were classified as low frailty (mFI5 < 2). High frailty patients had significantly higher Charlson Comorbidity Index compared to the low frailty group (5.1 ± 2.4 vs. 2.7 ± 2.3, *p* = 0.001). The high frailty group had significantly lower pain intensity (PROMIS‐3a: 55.5 ± 12 vs. 47.3 ± 12.5, *p* = 0.001) compared to the low frailty group. There was no significant difference in function (LEFS: 45.3 ± 20.1 vs. 44.5 ± 24.4, *p* = 0.42), psychological distress (SRQ‐20: 3.4 ± 3.5 vs. 2.9 ± 3, *p* = 0.19), or resilience (CD‐RISC: 7.2 ± 1.2 vs. 7 ± 1.4 *p* = 0.24) between low and high frailty groups.

**Conclusions:** Patient‐oriented multidisciplinary care and rehabilitation can help optimize limb salvage patients to perform at their maximum societal function.

## 21 118

### 21.1 Final Debridement Cultures Before Free Tissue Transfer Do Not Predict Limb Salvage Outcomes

#### 21.1.1 Joshua Carreras; Karen Evans, MD; Ryan Lin, MD; Rachel Rohrich; Sahil Sharma

21.1.1.1

**Background:** Chronic wounds are serially debrided prior to lower extremity (LE) free tissue transfer (FTT). Qualitative cultures taken after these debridements may guide clinical decisions. This study evaluates the impact of qualitative final debridement culture results LE FTT outcomes.

**Methods:** A retrospective chart review of patients receiving LE FTT from July 2011 to June 2024 was conducted.

**Results:** 345 LE FTT had final debridement culture data. The median Charlson Comorbidity Index was 4 (IQR: 3), reflecting prevalent rates of diabetes (55.9%), peripheral vascular disease (54.2%), chronic kidney disease (11.1%), and end‐stage renal disease (5.5%). Final debridement cultures were positive in 143 cases (41.4%). 43 wounds (12.5%) were polymicrobial. Flap survival rate was 96.8% and, by a median follow up time of 10.6 (IQR: 22.7), major limb amputation occurred in 40 cases (11.6%). Culture characteristics that conferred higher risk for amputation on univariate analysis included a bacterial load of “rare” (OR: 8.1, *p* = 0.013) and methicillin‐resistant Staphylococcus aureus (MRSA; OR: 3.7, *p* = 0.039). Upon multivariate analysis adjusting for all significant co‐variates to amputation, these variables did not remain significant. Rather, osteomyelitis on the date of surgery (OR: 3.1, CI: 1.2, 8.0, *p* = 0.018), more debridements before FTT (OR: 1.4, CI: 1.0, 1.8, *p* = 0.037), and an occluded posterior tibial artery (OR: 52.5, CI: 1.0‐6.1, *p* = 0.042) independently increased odds of amputation.

**Conclusions:** The limited predictive value found of the final debridement culture reinforces the capacity of healthy vascularized tissue to promote limb salvage, even in the presence of soft tissue infection.

## 22 119

### 22.1 Establishing a Guideline‐Based Multidisciplinary Diabetic Foot Programme: A 100‐Day Review of Clinical Characteristics and Outcomes

#### 22.1.1 Nur Faezah Binti Sani; Sadhana Chandrasekar, MD; Hui Xian Jaime Lin, MD; Kaamini Ravindran Pillay, MD; Yanli Shao

22.1.1.1

**Background:** Woodlands Health, a newly established hospital in Northern Singapore, introduced a pioneering multidisciplinary Diabetic Foot Programme (DFP) to provide comprehensive care for patients with diabetic foot ulcers (DFU). The program includes both inpatient care and a rapid‐access outpatient clinic. We aim to characterise patients in our DFP and track their clinical outcomes for the first 3 months. As a new program, the data analysis will offer insights for care and service improvements.

**Methods:** The is a descriptive statistical analysis of all patients within WH DFP between 2 May 2024 to 31 July 2024. Over the first 100 days of operation, WH DFP managed 106 patients. Inclusion criteria are patient above 21 years old, with pre‐existing diabetes mellitus and foot ulcers distal to the malleolus. A descriptive statistical analysis was conducted to evaluate clinical characteristics and outcomes.

**Results:** The mean age was 63.4 years, with a male predominance. Most patients had long‐standing diabetes and poor glycaemic control, leading to high rates of macrovascular and microvascular complications. Of the patients, 61.3% required inpatient management, 25.5% underwent revascularisation, and 34.9% had DFU‐related surgeries. Nearly 30% of patients were at medium to high risk of amputation based on the WIfI score. Minor lower extremity amputations (LEA) occurred in 15.1%, and 4.7% required major LEA. The 30‐day mortality rate was 3.8%, and the average length of stay was 15.7 days. The time to revascularisation was 4.4 days, and the time to DFU‐related surgery was 4.2 days. Wound healing was documented in 34.8% of patients, with a healing time of 63.2 days.

**Conclusions:** Our structured, multidisciplinary diabetic limb salvage program demonstrated favorable limb salvage outcomes despite high amputation risk. The early outcomes of the DFP highlight the effectiveness of early medical optimization, infection control, revascularization, and active wound care. However, challenges in patient adherence emphasize the need for further strategies to improve long‐term engagement and outcomes. Future studies with extended follow‐up and subgroup analyses are warranted to refine predictive models and optimize DFU care.

## 23 120

### 23.1 Beyond the Operation: The Role of Social Vulnerability in Recovery after Major Lower Extremity Amputation

#### 23.1.1 Karen Evans, MD; Sami Ferdousian, MS; Ryan Lin, MD; Rache Rohrich; Isabel Snee

23.1.1.1

**Background:** Major lower extremity amputations (MLEA), including below‐the‐knee amputation (BKA) and above‐the‐knee amputation (AKA), profoundly affects patients’ quality of life. The Social Vulnerability Index (SVI) quantifies the impact of demographic and socioeconomic factors on resilience to community‐based disasters, with higher numbers corresponding to more vulnerability and lower numbers corresponding to lower vulnerability. This study investigates the relationship between SVI and post‐amputation patient‐reported outcome measures in those with chronic lower extremity wounds undergoing MLEA.

**Methods:** From June 2022 through September 2024, a cross‐sectional study was performed at a tertiary wound care center for MLEA patients, who were stratified into low, average, and high SVI groups. PROMs were collected at 1‐, 3‐, 6‐month and 1‐, 3‐, and 5‐year intervals, including psychological distress (SRQ‐20), functionality (LEFS), pain (PROMIS‐3a), and resilience (CD‐RISC). Demographic, socioeconomic, and clinical data were analyzed, and statistical comparisons were performed between SVI groups.

**Results:** Ninety patients completed 201 surveys. High SVI patients lived closer to the hospital (median 14.2 miles) compared to low SVI (28.3 miles) (p = 0.006). PROMIS‐3a scores revealed higher pain levels in low SVI patients (52.8 ± 15.2) compared to average (46.5 ± 11.6) and high SVI patients (50.1 ± 13.9) (p = 0.027). LEFS scores indicated higher functionality in high SVI patients (43.9 ± 21.9) compared to low (32.6 ± 17.6) and average SVI (34.5 ± 16.5) (p = 0.003). SRQ‐20 and CD‐RISC scores showed no significant differences between groups.

**Conclusions:** High SVI patients demonstrated better functional recovery, potentially influenced by closer proximity to hospital rehabilitation services. Conversely, low SVI patients reported higher pain levels. These findings underscore the importance of integrating social determinants of health into postoperative care strategies to optimize recovery and patient satisfaction.

## 24 121

### 24.1 Impact of a Nitric Oxide‐Generating Wound Dressing in Diabetic Foot Ulcers Segmented by Infection Status and Wound Duration: Post‐Hoc Analysis

#### 24.1.1 Michael Edmonds, MD; Alan Horner, PhD; Chris Manu, MD; Daniel Metcalf, PhD

24.1.1.1

**Background:** Nitric oxide (NO) within a wound dressing represents a promising agent for use in DFU treatments due to its antimicrobial properties. Here we evaluate the effectiveness of a prototype NO‐generating dressing (NOGD) on the healing rates of DFUs segmented by infection status and wound duration in a post‐hoc analysis of the ProNox1 randomized controlled trial [1].

**Methods:** The impact of infection status and wound duration on the number of DFUs healed in the intention to treat (ITT) cohort were assessed.

**Results:** Of the 149 ITT DFUs, 38/74 (NOGD arm) and 39/75 (SoC) were infected and/or returned positive culture at baseline. At week 12, healing rates in infected/contaminated DFUs were 36% (NOGD) vs 21% (SoC) and 35% vs 30% for non‐infected DFUs. Healing trajectories continued only in the NOGD arm, with 46% healed by week 16.

In wounds ≤ 12 weeks in duration, healing at week 12 was 47% (NOGD) and 24% (SoC). Healing rates increased in week 16 follow‐up in both groups with 56% (NOGD) and 28% (SoC) healed. In DFUs > 12 weeks of duration, healing rates were the same for both arms: 25% in the study period and 30% at final follow‐up. When DFUs > 12 weeks of duration were further segmented by infection/contamination, healing rate was 35% (NOGD) at final follow‐up compared with 18% (SoC).

**Conclusions:** This sub‐analysis of the ProNox1 study data demonstrated the ability of NOGD to improve the DFU healing rate in infected/contaminated (at‐risk) DFUs and DFUs of < 12 weeks, compared with SoC.

## 25 122

### 25.1 Use of Dermal Replacement Scaffold as an Adjunct in Split‐thickness Skin Grafting: A Retrospective Case Series

#### 25.1.1 Bryan Barbato, DPM; Ashish Bjiu, DPM, PGY‐2; Bradley Levitt, DPM; Jonathan Westerveld, DPM, PGY‐2

25.1.1.1

**Background:** Soft tissue defects in the foot and ankle pose a difficult challenge for podiatric surgeons. Split‐thickness skin grafting (STSG) provides coverage of defects with a high success rate. While split thickness skin grafting is the gold standard for coverage, recipient sites can take weeks to mature to accept STSG autograft, especially if bone or tendon is exposed. We describe a one‐step technique using a dermal replacement scaffold that allows for early skin grafting in this case series.

**Methods:** A retrospective analysis was performed on seven patients with diabetic foot infections undergoing simultaneous dermal replacement scaffolding and split‐thickness skin grafting in our center from February 1, 2024 to October 16, 2024. Data including age, BMI, and most recent A1c were collected.

The procedure was performed under monitored anesthesia care and local anesthetic. The involved foot was aggressively debrided and irrigated to achieve a clean and healthy wound bed. After hemostasis was achieved an acellular dermal matrix was secured over the wound. An autologous split‐thickness was then harvested from the ipsilateral extremity, meshed to 1.5:1 ratio, and fixed over the acellular dermal matrix. A bolster dressing or a wound vac was then applied over the surgical site and set to 80 mmHg of negative pressure. Wound dressing were replaced at 5 days and site evaluated for complications. Dressings were replaced every three days until the wound was closed.

Patients were followed for 9 months post‐procedure. The surgical sites were evaluated for wound healing progression, graft failure, and recurrent infection

**Results:** The average age for the patients were 63.1 (range 58‐67). The average BMI was 32.0 (25.7‐38.9). The average hemoglobin A1c was 6.7% (5.1‐9.1). 7 of the 7 patients had successful epithelialization of their graft site at final follow up. 1 patient had their initial ADM/STSG fail due to excess moisture causing graft failure. This patient underwent traditional STSG and healed uneventfully. 0 patients developed recurrent ulceration to the operative foot.

**Conclusions:** A one step application of a dermal replacement scaffold and a meshed autologous split thickness skin graft provides effective coverage of diabetic foot wounds. This method allows for early reconstruction of complex wounds by bypassing the time between initial debridement and final grafting. Our results show an acceptable success rate in a delicate patient population.

## 26 123

### 26.1 Vein Arterialization for No‐Option Limb Salvage

#### 26.1.1 Eleanor Dunlap, CRNP, RN; Suzanna Fitzpatrick, CRNP, RN; Shannon Hawkins, RN; Khanjan Nagarsheth, MD

26.1.1.1

**Background:** Diabetes mellitus (DM) increases the risk of chronic limb‐threatening ischemia (CLTI) 2‐4 fold in peripheral arterial disease (PAD) patients. Successful tissue closure of diabetic foot ulcerations (DFU) requires inline arterial flow for healing. In patients with severe distal outflow occlusions where traditional revascularization isn't possible, venous arterialization (VA) offers a promising alternative for limb salvage.

**Methods:** A retrospective chart review examined four patients with no‐option Rutherford IV‐V chronic limb ischemia treated with DVA in 2024. Data collection included demographics, medical history, preoperative diagnoses, clinical symptoms, and imaging, with postoperative follow‐up through chart review.

**Results:** Four patients with no‐option CTLI and DFU were identified including two females (50%), with a mean age of 69 years (range 60‐75). All patients had failed prior revascularization and DFU. Common comorbidities included prior stroke (75%), hypertension (50%), and lipoprotein A deficiency (25%). Treatment was equally split between superficial and deep VA. The average time from VA to surgical debridement was 9 weeks (range 7‐10), and from debridement to closure was 14 weeks (range 8‐20), with one patient's treatment ongoing. All patients received weekly wound care from vascular or podiatric providers.

**Conclusions:** Venous arterialization can be used to improve inline flow to the wound in patients with no‐option CLTI and DFU. Waiting for appropriate wound demarcation prior to surgical debridement and aggressive wound care can help salvage limbs.

## 27 124

### 27.1 Application of Extracorporeal Membrane Oxygenation Perfusion Technology in the Treatment of Diabetic Foot Ulcer

#### 27.1.1 Lei Gao, MD, PhD; Jiangning Wang, MD, PhD

27.1.1.1

**Background:** Diabetic foot ultimately leads to amputation due to severe limb ischemia. Extracorporeal Membrane Oxygenation Perfusion Technology(ECMOPT) utilizes the principle of venous‐arterialization to improve blood supply to the ischemic limb, accelerate wound healing, and prevent amputation.

**Methods:** After applying the inclusion criteria and exclusion criteria, We retrospectively evaluated 69 patients with diabetic foot ulcer admitted from August 2020 to August 2024.The patients were grouped according to whether they received Extracorporeal Membrane Oxygenation Perfusion Technology; experimental group: 24 patients, control group: 45 patients. Foot microcirculation was evaluated by measuring the percutaneous oxygen partial pressure (TcPO2), infrared thermography (IRT). Wound healing time and ulcer recurrence rate 3 months after discharge were compared between the groups.

**Results:** TcPO2 and IRT values in the experimental group differed significantly compared with the control group(p < 0.05). Foot ulcer healing time in the experimental group was shorter than that in the control group (p < 0.05), and the recurrence rate after 3 months in the experimental group was lower than that in the control group (4/24, 16.7% vs 14/45,31.1%, respectively, *p* < 0.05).

**Conclusions:** Extracorporeal Membrane Oxygenation Perfusion Technology is a technique that effectively improves lower limb blood supply by utilizing the principle of venous‐arterialization.

## 28 125

### 28.1 Collagen Wound Matrix with PHMB: An Antimicrobial Barrier for Chronic and Complex Wounds

#### 28.1.1 Justin Avery, PhD; Kelly Kimmerling, PhD; Katie Mowry, PhD; Rami Nasrallah

28.1.1.1

**Background:** Diabetic patients often have complications including immune dysfunction, microvascular impairment, and diabetic foot ulcers (DFUs). DFUs significantly increase the risk of infection and, in severe cases, may lead to lower extremity amputation. Biofilms are present in over 90% of chronic wounds and can reform within 24 h, underscoring the critical need for timely biofilm management and wound closure. Herein, we describe two in vivo MRSA‐infected wound models evaluating a collagen wound matrix embedded with polyhexamethylene biguanide (PCMp *) as a protective barrier against biofilm reformation and a matrix to support wound healing.

**Methods:** A large dermal wound porcine model and an acute fracture complex wound canine model were used to study MRSA‐infected wounds.

**Results:** In the dermal wound study, diminished bioburden and increased wound closure were observed compared to untreated wounds; however, treatment until closure was critical, as removal of PCMP barrier resulted in a resurgence of bioburden before closure. In the canine model, significantly improved healing scores on days 3 and 7 were observed, along with reduced bacterial load and inflammation compared to control animals. Interestingly, both studies found consistent results of gene modulation of immune/inflammatory markers, proteases, and extracellular matrix collagens in PCMp ‐treated wounds, indicative of supporting a native wound healing response.

**Conclusions:** These findings show that PCMP provides a barrier, reduces biofilm reformation, and supports innate wound progression. This approach offers a promising solution to support wound healing in complex wound scenarios.

## 29 126

### 29.1 Limb Salvage Versus Amputation: A Comparative Analysis of Functional Gait Patterns

#### 29.1.1 Meghan Currin; Luke Llaurado; Rajan Negassa; Kishan Shah; Jie Jung Shih, MS

29.1.1.1

**Background:** Transmetatarsal amputation (TMA) and free tissue transfer (FTT) are limb salvage (LS) techniques that are considered before below‐knee amputation (BKA) for patients with chronic wounds. There is limited research on whether these patients’ lower extremity function support their quality of life. Thus, this study aims to compare spatiotemporal gait parameters between patients undergoing LS vs BKA.

**Methods:** A single‐centered, cross‐sectional study was conducted from June 2021‐September 2024. Patients with TMA, FTT, and BKA were identified, and gait data was compared between groups.

**Results:** A total of 67 patients were identified: 26(38.8%) LS and 41(61.2%) BKA. Patient demographics and comorbidities were similar between groups. Patients who underwent BKA demonstrated significantly lower mid‐swing elevation (LS: 1.91 ± 2.11 vs BKA: 0.74 ± 0.91, *p* = 0.002), relied less on single limb support (35.93 ± 2.09 vs 34.36 ± 3.86, *p* = 0.031), and took shorter steps (0.82 ± 0.41 vs 0.67 ± 0.41, *p* = 0.030). The BKA group demonstrated improved balance and stability compared to the LS group, as demonstrated by higher RMS sway (0.24 ± 0.18 vs 0.16 ± 0.10, *p* = 0.009).

**Conclusions:** Nontruamtic BKA patients exhibit compromised limb mechanics compared to LS patients, with less upward motion during the gait cycle and less time spent on their amputated leg. The increased postural stability observed may be attributed to compensatory mechanisms as patients navigate with prostheses. Further investigation in this under researched population is warranted.

## 30 127

### 30.1 Assessing Treatment Efficiency in Diabetic Foot Ulcers: Processing for Retention Versus Lamination: A Retrospective Analysis

#### 30.1.1 Robert Frykberg, DPM, MPH; Toni‐Ann Martorano, MS; Zwelithini Tunyiswa; Wendy Weston, PhD

30.1.1.1

**Background:** Diabetic foot ulcers are a severe complication in diabetic patients that significantly impact healthcare systems and patient quality of life, often leading to hospitalization and amputation. Traditional Standard of Care (SOC) treatments are inadequate for many patients, necessitating advanced wound care products like human placental membranes. These products are intended as covering for acute and chronic wounds. We conducted a retrospective analysis to compare the effectiveness of two human placental membrane products, retention‐processed (RE‐AC) and lamination‐processed (L‐AC) in managing diabetic foot ulcers.

**Methods:** The study collected retrospective observational data from electronic health records of patients treated at three outpatient wound care centers. Patients were categorized into two cohorts based on the treatment received. Key metrics included wound size progression and the number of product applications. The analysis employed Bayesian regression and Hurdle Gamma Analysis of Variance (ANCOVA) models.

**Results:** Even with a higher starting wound area, results indicated that RE‐AC achieved a higher expected Percent Area Reduction (xPAR) compared to L‐AC at 12 weeks. Although L‐AC was slightly more effective in complete wound closure, RE‐AC required 27% fewer applications and 14% fewer treatment days, suggesting greater efficiency in general wound closure.

**Conclusions:** RE‐AC, as a wound covering, offers overall better treatment efficiency, especially in reducing the frequency of applications. This efficiency can lead to improved patient comfort, reduced treatment costs, reduced rate of amputation, and optimized resource utilization in healthcare settings.

## 31 128

### 31.1 Understanding Preferences on Limb Salvage for Diabetic Foot Ulcers: A Discrete Choice Experiment

#### 31.1.1 Justin Kim, MD; Alison Lydecker, MPH; Nathan O’Hara, PhD; Gwen Robinson, MPH; Mary‐Claire Roghmann, MD

31.1.1.1

**Background:** Diabetic foot ulcers (DFUs) are a severe complication of diabetes, often leading to infections and amputations. Limb salvage aims to preserve the lower extremity, but the complexity of care and uncertainty of healing can delay patients’ return to normal activities. This study aimed to understand Veterans’ preferences regarding limb salvage for DFUs using a discrete choice experiment (DCE).

**Methods:** A DCE was conducted with 98 Veterans with diabetes at the Baltimore VA Medical Center. Participants were presented with 10 choice sets involving different levels of post‐recovery mobility, amputation levels, and future surgery risks. These attributes and levels were developed through literature review and semi‐structured interviews. Data were analyzed using a multinomial logit model to estimate the utility of each attribute level and assess preference heterogeneity.

**Results:** The study population was predominantly older (mean age 69 years), Black (61%), and male (94%). Mobility was the most important attribute (relative importance 53%), followed by amputation level (30%) and future surgery risk (18%). Mobility was highly valued, with significant utility differences between walking unaided and needing a wheelchair/scooter. Veterans were willing to accept higher amputation levels to improve mobility. Preferences varied by amputation history, though differences were not statistically significant.

**Conclusions:** Mobility is a critical factor for Veterans with DFUs, outweighing concerns about amputation level and future surgical risks. Post‐recovery mobility should be a focus of shared decision making between Veterans and their health care providers.The study's limitations include its single‐site setting and a study population that did not currently have a DFU. There is a need for broader research. Understanding patient preferences through DCE can inform more patient‐centered approaches to DFU management, potentially improving outcomes and satisfaction.

## 32 129

### 32.1 Gait Parameters Following Non‐Traumatic Minor and Major Amputations: A Comparative Analysis For Functional Limb Salvage

#### 32.1.1 Holly Shan; Emery Steinberg

32.1.1.1

**Background:** Limb salvage surgeons are often faced with choosing between a transmetatarsal amputation (TMA) and below‐knee amputation (BKA) when there is unsalvageable foot pathology. Although TMA, a lower level amputation that has greater potential for return to ambulation, the chances of reoperation to a higher level of amputation is greater, thus exposing a patient already with high comorbidities to more high‐risk surgeries. This study investigates functional outcomes of ray amputations, TMA, and BKA through comparative gait analysis. Updated abstract from 2024.

**Methods:** From December 2021‐January 15 2025, adult patients who could safely ambulate unassisted and without pain, without open wound, and without previous lower extremity surgery in the previous three months were offered participation. Patients with ray amputation, TMA, or BKA were included in this analysis. Charleston Comorbidity Index (CCI) scores were found through chart review and level of amputation were verified through x‐ray review. Participants completed a standardized protocol with wearable sensors, completed a 120‐second walk test at a self‐selected pace, and a 30‐second Romberg (sway) test. We analyzed data provided from the Motility Lab software. Data was analyzed using ANOVA with significance defined as *p* < 0.05

**Results:** Of the 375 patients in the gait database, 72 patients received an amputation (Digit n=15, Ray n=15, TMA n=13, BKA, n=25). Average age was 60.9 1.1, BMI 30.70.67, CCI score 4.80.3, and 78 (71%) were male. There were no differences in gait parameters across all amputation levels in gait speed, elevation mid‐swing, step duration, cadence, single limb support, double limb support, and sway.

**Conclusions:** Ambulation is important for quality of life in limb salvage patients, and no clear guidelines exist in on how to preserve functionality based on level of amputation. To the authors’ knowledge, this is the first study to use gait as a measure of functionality to compare multiple levels of amputation. Gait parameters did not differ between patients receiving TMA and BKAs.

## 33 130

### 33.1 Enhancing Limb Salvage Success in Racially and Ethnically Underserved Groups: The Benefits of a Highly Integrated Multidisciplinary Approach

#### 33.1.1 Rajan M. Negassa, BS; Nisha J. Gupta, MS; Rachel N. Rohrich, BS; Meghan E. Currin, BA; Sami Ferdousian, MS; Omar Allam, MD; Paris Butler, MD; Richard C. Youn, MD; Christopher E. Attinger, MD; Cameron M. Akbari, MD; Karen K. Evans, MD

33.1.1.1

**Background:** In the setting of lower extremity (LE) limb salvage, prior studies have highlighted discrepancies in postoperative outcomes and amputation rates in racial minority patients, however this analysis has not been evaluated in the setting of free tissue transfer (FTT) for chronic, non‐healing wounds. This study seeks to analyze outcomes by race in our institution's 13‐year experience with LE FTT.

**Methods:** All LE FTTs performed between 2011 and 2024 were retrospectively reviewed. Demographics, socioeconomic characteristics, operative details, and outcomes were recorded.

**Results:** 335 FTTs were included for analysis: 49% White (n=164), 45.4% Black (n=152), and 5.7% Hispanic/Asian (n=19). Significant differences were observed in age (p < 0.01), BMI (p = 0.008), rates of diabetes (p = 0.004), end‐stage renal disease (p = 0.023), and peripheral neuropathy (p = 0.028), with Black patients being the most comorbid. Median income ($97,694 vs. $124,371 vs. $107,542, *p* < 0.001), poverty rates (10.6% vs. 6.7% vs. 7.7%, *p* < 0.001), and percentage of individuals with a Bachelor's degree or higher (27.2% vs. 32.6% vs. 30.1%, *p* = 0.001) were lowest among Black patients. There were no significant differences in any flap complications, postoperative below‐knee amputations, ambulatory status at follow‐up, or mortality rates.

**Conclusions:** With an integrated multidisciplinary approach to limb salvage, favorable outcomes can be achieved, regardless of race. By optimizing patients and ensuring long‐term follow‐up, the effects of socioeconomic characteristics can be mitigated.

## 34 131

### 34.1 Next‐Generation Sequencing vs. Conventional Culture: A Paradigm Shift in Diagnosing Diabetic Foot Ulcer Infections

#### 34.1.1 Seung‐Kyu Han, MD, PhD; Gordon Lee, MD, FACS; Sik Namgoong, MD, PhD

34.1.1.1

**Background:** Accurate identification of wound‐site microbiota is critical for optimizing antimicrobial therapy and improving prognosis in diabetic foot ulcers (DFUs). Conventional culture‐based methods have limitations in detecting anaerobes and fastidious organisms, whereas 16S rRNA next‐generation sequencing (NGS) has emerged as a more comprehensive approach. This study compares conventional culture and 16S rRNA NGS to evaluate their predictive value for DFU treatment outcomes.

**Methods:** Wound‐site samples from 47 DFU patients treated at Korea University Guro Hospital between February and November 2021 were analyzed using both conventional culture and 16S rRNA NGS. The primary outcome was healing status, assessed by the SINBAD score and amputation status. Secondary outcomes included wound‐site ischemia and infection control. Microbial diversity was quantified using the Shannon and Simpson indices, and species concordance between methods was evaluated.

**Results:** NGS identified a broader spectrum of microbial species (Shannon index: 1.369 ± 0.755; Simpson index: 2.987 ± 1.383) compared to conventional culture (Shannon index: 0.693; Simpson index: 1.269). A total of 16 anaerobic species were commonly detected by both methods. The most significant discordance in microbial identification was observed in patients with SINBAD scores =3 (40.79%), particularly in those with poor infection control despite the absence of ischemia (85.22%). Key pathogenic species associated with poor prognosis, including Bacteroides fragilis, Streptococcus agalactiae, Staphylococcus aureus, and Streptococcus constellatus, were more frequently identified using NGS than culture.

**Conclusions:** Early findings suggest that 16S rRNA NGS may enhance diagnostic accuracy in diabetic foot infections. Larger studies are needed to confirm its clinical utility.

## 35 132

### 35.1 Clostridium Difficile Induced Coagulopathy: Implications for Microsurgery in the Setting of Limb Salvage

#### 35.1.1 Roxana Azimi, MD; Karen Evans, MD; Alexandra Junn, MD; Karen Li; Rachel Rohrich

35.1.1.1

**Background:** Studies have demonstrated a correlation between Clostridium difficile infection (CDI) and hypercoagulability. This study evaluates impact of CDI in patients undergoing lower extremity (LE) free tissue transfer (FTT) and quantifies its impact on microsurgical outcomes.

**Methods:** A retrospective cohort study of patients receiving LE FTT from July 2011 to June 2024 was conducted. Patients who tested positive for C. difficile within 15 days of their FTT were identified and compared to those who did not.

**Results:** A total of 356 LE FTT were performed. Six patients (1.7%) contracted CDI. Groups had similar comorbidity and wound profiles. Flap takeback occurred at significantly higher rates in the CDI group (66.7% vs. 6.3%; *p* < 0.001), as did microvascular pedicle thrombosis (33.3% vs. 3.1%; *p* = 0.017). Flap complications were also significantly higher in the CDI group (100.0% vs. 27.7%; *p* = 0.001), specifically partial flap necrosis (50.0% vs. 3.1%; *p* = 0.001) and infection (66.7% vs. 12.6%; *p* = 0.004). By a median follow‐up of 15.7 months, major LE amputation rates were similar between CDI and Non‐CDI groups (33.3%, n=2 vs. 12.6%, n=44). Multivariate regression models adjusting for statistically and clinically significant co‐variates demonstrated CDI as an independent risk factor for flap takeback (OR: 46.4, 95% CI: 7.2‐297.36, *p* < 0.001) and microvascular thrombosis (OR: 26.4, CI: 3.5‐197.3, *p* = 0.001).

**Conclusions:** Perioperative infection with C. diff may independently increase immediate microvascular thrombolytic risk in LE FTT. Plastic surgeons should be aware of the immediate microvascular risks associated with C. Diff infection. Further research is required to fully understand the clinical management of this population.

## 36 133

### 36.1 Understanding the Prevalence of Medial Arterial Calcification Among Complex

#### 36.1.1 Reconstructive Patients: Insights from a Decade of Experience at a Tertiary Limb Salvage Center

Christopher Attinger, MD; Nicole Episalla, MD; Sami Ferdousian, MS; Ryan Lin, MD; Rachel Rohrich

36.1.1.1

**Background:** Medial arterial calcification (MAC), a distinct form of vascular pathology frequently coexisting with peripheral arterial disease (PAD), poses unique challenges in limb salvage among patients with diabetes, chronic kidney disease, and end‐stage renal disease. This study examines the incidence of MAC and its impact on limb salvage outcomes over a decade of experience at a tertiary limb salvage center.

**Methods:** A retrospective review of all lower extremity (LE) reconstructions using local flap (LF) or free tissue transfer (FTT) performed from July 2011 to September 2022 was conducted. Patients were classified into MAC and No MAC groups based on pedal radiography evaluation using the Ferraresi MAC scoring system. Primary outcomes were major lower extremity amputation (MLEA), the need for postoperative vascular intervention, major adverse limb events (MALE; defined as the composite of any reoperation, MLEA, or postoperative revascularization attempt), and mortality.

**Results:** During the study period, a total of 430 LE reconstructions were performed with LF or FTT. A total of 323 cases (75.1%) demonstrated no MAC while the remaining 107 (24.9%) demonstrated MAC. The MAC group exhibited significantly higher rates of diabetes, PAD, and renal disease. By a follow‐up duration of 17.0 (IQR: 33.9) months, the MAC group demonstrated a significantly higher rate of MLEA (24.3% vs. 13.0%, *p* = 0.006), postoperative vascular intervention (23.4% vs. 8.7%, *p* < 0.001), MALE (57.0% vs. 25.7%, *p* < 0.001), and mortality (28.0% vs. 9.9%, *p* < 0.001). Multivariate analysis identified MAC as independently predictive of MALE (OR: 1.8, CI: 1.1‐3.0, *p* = 0.033).

**Conclusions:** MAC is prevalent among surgical candidates for limb salvage. Patients with MAC represent a significant medical and reconstructive challenge. Radiographic screening for MAC should be considered in all limb salvage candidates with LE wounds, especially in those with diabetes and kidney disease. Assessing MAC is important to better evaluate risk factors and surgical options so as to optimize outcomes.

## 37 134

### 37.1 Endovascular Intervention Prior to Lower Extremity Free Tissue Transfer Provides Effective and Durable Limb Salvage in a 13‐Year Analysis

#### 37.1.1 Karen Evans, MD; Sami Ferdousian, MS; Ryan Lin, MD; Rachel Rohrich; Elonay Yehualashet

37.1.1.1

**Background:** Free tissue transfer (FTT) in patients with peripheral arterial disease (PAD) and lower extremity (LE) tissue loss is a complex option for reconstructive limb salvage. Previous studies have demonstrated the importance of preoperative arteriography for success; however, the role of preoperative percutaneous endovascular intervention (PEI) has not been comprehensively studied. Thus, this study evaluates the role of preoperative PEI in LE FTT for the management of chronic, non‐healing wounds.

**Methods:** A retrospective review of all LE FTT between July 2011 and July 2024 was conducted. Patients were classified into two groups based preoperative PEI treatment (“PEI” group vs. “Non‐PEI” group).

**Results:** A total of 338 LE FTT were performed in 329 patients, of which 61 (18.5%) underwent preoperative PEI. The median age was 57 (IQR: 17) years. Prevalent comorbidities included diabetes (55.3%), PAD (38.0%), and chronic kidney disease (10.9%). At a median follow‐up of 12.2 months, rates of ipsilateral amputation (12.2%) were not statistically different between PEI and Non‐PEI groups. However, the PEI group experienced a significantly higher incidence of any postoperative complication (46.03% vs. 25.3%, *p* = 0.001), including increased rates of partial flap necrosis (9.52% vs. 1.84%, *p* = 0.001) and flap hematoma (11.29% vs. 4.40%, *p* = 0.034). Mortality rates did not differ significantly between groups.

**Conclusions:** Despite significant differences in baseline comorbidities, patients requiring PEI achieved limb salvage and flap success rates comparable to those without the need for such intervention. Preoperative vascular optimization via PEI is an effective tool to expand reconstructive options for patients with advanced PAD.

## 38 135

### 38.1 The Use of Local Flaps For Last‐Option Limb Salvage in Patients with One Vessel Run‐Off

#### 38.1.1 Christopher, MD; Sami Ferdousian, MS; Karen Li; Ryan Lin, MD; Rachel Rohrich

38.1.1.1

**Background:** Local flaps are a mainstay for small to medium‐sized defects. We have found them to be useful in polymorbid patients with advanced arterial disease that precludes free flap reconstruction. This study presents our experience using local flaps for foot and ankle defects in patients with a single vessel run‐off (VRO).

**Methods:** A review of local flaps of the foot and ankle between January 2010 and November 2022 was conducted. VRO was defined as the number of patent tibial vessels (posterior tibial, anterior tibial, and peroneal). Patients with 1‐VRO were included in this study.

**Results:** 18 patients were identified. Mean age was 68.2 ± 11.5 years and mean Charlson Comorbidity Index was 7.8 ± 1.9, conferring a 0% estimated 10‐year survival rate. Diabetes (77.8%), chronic kidney disease (72.2%), and end‐stage renal disease (44.4%) were prevalent. In 10 patients (55.6%), a preoperative vascular intervention was performed, resulting in an increase in VRO from 0 to 1 in 7 patients. Mean wound size was 51.1 ± 28.6 cm2. Flap blood supply included pedicled (61.1%) and random pattern (38.9%). By a median follow‐up period of 27.9 (IQR: 190.5) months, limb salvage rate was 61.1% (n=11) and mortality rate was 27.8% (n=5).

**Conclusions:** Local flaps may be successfully used as a last reconstructive option in patients with large wounds and extremely disadvantaged vascular status. Preserving limb length in this extremely comorbid population is of utmost important to maintain mobility. Close work with a vascular surgery team is essential to manage this population.

## 39 136

### 39.1 Is the Free Medial Sural Artery Perforator Flap A Reliable Choice For Reconstruction of Chronic, Atraumatic Lower Extremity Wounds?

#### 39.1.1 Meghan Currin; Nicole Episalla, MD; Karen Evans, MD; Karen Li; Rachel Rohrich

39.1.1.1

**Background:** The medial sural artery perforator (MSAP) flap is a thin and pliable flap that can be raised as either a free or pedicled fasciocutaneous flap in lower extremity (LE) reconstruction. The utility of the MSAP flap has not yet been described in the chronic wound population. The aim of this study is to present our institution's experience using the free MSAP flap exclusively for the reconstruction of chronic LE wounds.

**Methods:** A retrospective review of patients receiving LE free tissue transfer with an MSAP flap was performed.

**Results:** Ten patients received free fasciocutaneous LE MSAP flaps. The mean age and body mass index was 49.7 ± 16.4 years and 28.0 ± 4.9 kg/m2, respectively. Notable comorbidities were diabetes (n=4, 40.0%) and a history of cancer (n=4, 40.0%). Average defect size was 25.6 ± 11.2 cm2. All 10 flaps were successful (n=10, 100.0%). Takeback due to concern for venous congestion between postoperative day 0 and 7 occurred in two cases (n=2, 20.0%), both of which were salvaged. Other complications included dehiscence (n=2, 20.0%), infection (n=1, 10.0%), and partial flap necrosis (n=2, 20.0%). Both patients with partial flap necrosis required leech therapy and prolonged hyperbaric oxygen for complete wound healing. Limb salvage was 100.0% (n=10).

**Conclusions:** The MSAP flap has favorable microsurgical outcomes however it has been our experience that it has high recipient site complications including dehiscence, infection, and partial flap necrosis that require extensive postoperative care, healthcare resource utilization, and additional postoperative visits. The MSAP flap might be a useful flap for the chronic wound population, but further studies are needed to fully understand how reliable a choice for this complex population.

## 40 137

### 40.1 Prevalence of Popliteal Artery Variants in Free Tissue Transfer for Limb Salvage: A 12‐Year Vasculo‐Plastic Experience

#### 40.1.1 Karen Evans, MD; Sami Ferdousian, MS; Karen Li; Rachel Rohrich; John Rutland, MD

40.1.1.1

**Background:** Popliteal artery variants (PAVs) are anatomical deviations of the popliteal artery's branching pattern and should be considered in microsurgical planning for patients undergoing lower extremity (LE) free tissue transfer (FTT). However, there is a significant lack of FTT literature in this patient population. Thus, this study presents our 12‐year experience with LE FTT in patients with PAV.

**Methods:** Patients receiving LE FTT reconstruction from July 2011 to March 2024 were reviewed. Preoperative angiograms were reviewed by a single vascular surgeon, and the presence of PAV was identified and classified as IIIA, IIIB, or IIIC. Primary outcomes were flap success and limb salvage.

**Results:** A total of 339 LE FTT were performed in 331 patients. 32 patients (9.4%) had PAV, accounting for a total of 34 LE FTT. Class III‐A was the most common category (n=20, 58.8%) followed by III‐B (n=8, 23.5%) and III‐C (n=6, 11.7%). Median age and BMI were 63.5 (IQR: 22.5) years and 27.4 (IQR: 10.3) kg/m2. The median Charlson Comorbidity Index was 5 (IQR: 2.5), with prevalent rates of diabetes (n=18/32, 56.3%) and peripheral artery disease (n=16/32, 50.0%). Median wound area was 71.0 (IQR: 80.0) cm2. Flap success rate was 100% (n=34/34). At a median follow‐up of 12.8 (IQR: 22.6) months, limb salvage was 97.1% (n=33/34) and mortality was 6.3% (n=2/32).

**Conclusions:** In this large population of LE FTT, PAV occurs in almost one out of ten patients. Essential to flap success and limb salvage is appropriate preoperative vascular imaging with arteriography, as the presence of PAV changes microsurgical intraoperative planning and technical considerations.

## 41 138

### 41.1 Predictive Factors of Successful Salvage Following Takeback in the Setting of Lower Extremity Limb Salvage: Our Institution's 12‐Year Experience

#### 41.1.1 Meghan Currin; Karen Evans, MD; Sami Ferdousian, MS; Ryan Lin, MD; Rachel Rohrich

41.1.1.1

**Background:** Microvascular compromise requiring re‐exploration is a devastating complication following free tissue transfer (FTT), especially for the chronic lower extremity (LE) wound population who may face amputation following flap failure. Because microsurgical success rates are high, there is limited clinical data exploring patient and operative factors that contribute to successful flap salvage after takeback, especially in the heavily comorbid LE wound population. Thus, this study aims to identify factors that predict salvage after LE FTT takeback in the setting of limb salvage.

**Methods:** A retrospective chart review of LE FTT performed from February 2011 to June 2024 was performed. Patient characteristics, intraoperative details, and postoperative outcomes were collected. Flap takeback was defined as a return to the operating room for flap exploration due to concern for flap viability. Flaps that were salvaged were compared to flaps that were not.

**Results:** During the study period, 357 LE FTT were performed, of which 26 cases (7.2%) required takeback. A total of 19 (73.1%) flaps salvaged, while 7 (26.9%) were not. Although not statistically significant, the salvage group trended to be younger (52.6 ± 12.6 vs. 63.3 ± 12.0, *p* = 0.064) and have a lower BMI (28.2 ± 6.7 vs. 33.6 ± 6.8, *p* = 0.083). Median hospital length of stay was significantly higher in the salvage group (32, IQR: 10 vs. 23, IQR: 7; *p* = 0.017). In the total cohort, diabetes (65.4%), peripheral vascular disease (42.3%), and chronic kidney disease (23.1%) were prevalent, with no differences between groups. A total of 6 patients (24.0%) required vascular intervention to optimize distal perfusion prior to reconstruction (p =1.000). Median wound area trended to be larger in the salvage group (112, IQR: 48 vs 60, IQR: 37; *p* = 0.078). There was a higher incidence of flap monitoring devices used in the salvage group, notably the Cook Doppler (84.2% vs. 57.1%, *p* = 0.293) and ViOptix (52.6% vs. 14.3%, *p* = 0.178). The median time to takeback was 1 day (IQR: 7 days) in the salvage group and 5 days (IQR: 7 days) in the failure group (p = 0.280). By a final follow‐up of 8.1 (IQR: 9.6) months, 2 patients (7.7%) underwent major limb amputation (p = 0.474). Univariate analysis demonstrated that Cook Doppler use was significantly associated with higher odds of flap salvage (OR:11.3, CI: 1.4‐92.1, *p* = 0.023).

**Conclusions:** This study represents the largest series of LE FTT takeback in the setting of atraumatic limb salvage. Our results reinforce the importance of close monitoring and early intervention, which may contribute to successful flap salvage.

## 42 139

### 42.1 The Role of Local Flap Reconstruction for Limb Salvage in Patients with Moderate to Severe Medial Arterial Calcification

#### 42.1.1 Christopher Attinger, MD; Sami Ferdousian, MS; Ryan Lin, MD; Rachel Rohrich; Isabel Snee

42.1.1.1

**Background:** The medial arterial calcification (MAC) scoring system predicts adverse limb events. This study applies MAC scoring to patients undergoing local flap reconstruction.

**Methods:** Patients that underwent foot and ankle local flaps from January 2010 to November 2022 were reviewed. Radiographs were used to assigned MAC scores: absent (MAC= 0‐1), moderate (MAC=2‐3), or severe (MAC=4).

**Results:** 182 patients underwent local flap reconstruction: 104 (57.1%) absent MAC, 32 (17.6%) moderate MAC, and 46 (25.3%) severe MAC. Patients with severe MAC demonstrated significantly higher rates of diabetes mellitus (p = 0.001), end‐stage renal disease (p < 0.001), and peripheral neuropathy (p < 0.001), and more often required a vascular intervention before reconstruction (p = 0.001). Flap ‐related outcomes and rates of major limb amputation were statistically similar between MAC groups. By a median of 16.5 (IQR: 36.6) months, limb salvage was 84.1% and not independently associated with MAC on multivariable analysis. Postoperative vascular intervention (absent: 10.7% vs. severe: 28.1% vs. severe: 17.4%; *p* = 0.054), podiatric reoperation (absent: 35.6% vs. severe: 40.6% vs. severe: 56.5%; *p* = 0.056), and mortality (absent: 19.4% vs. severe: 34.4% vs. 32.6%; *p* = 0.102) were not independently associated with MAC on multivariable analysis.

**Conclusions:** Local flaps are a viable option in patients with MAC. If utilizing a vasculo‐plastic approach, severe MAC should not prevent limb salvage efforts via local flap reconstruction.

## 43 140

### 43.1 Do GLp ‐1 Medications Influence Lower Extremity Free Tissue Transfer Outcomes in Diabetic Limb Salvage Patients? A 13‐Year Retrospective Review

#### 43.1.1 Karen Evans, MD; Sami Ferdousian, MS; Ryan Lin, MD; Rachel Rohrich; Finn Tobias

43.1.1.1

**Background:** Glucagon‐Like Peptide 1 (GLp ‐1) medications are increasingly used for obesity and diabetes management. This study investigates the impact of GLp ‐1 medications on lower extremity (LE) free tissue transfer (FTT) outcomes in diabetic limb salvage patients.

**Methods:** A retrospective review of all LE FTT performed in diabetic patients from 2011 to 2024 was performed.

**Results:** 198 LE FTT were performed in 192 diabetic patients: GLp ‐1 medication was used by 21 patients (10.9%) at the time of FTT, with a median usage duration of 14.1 months (IQR: 27.7 months). GLp ‐1 usage significantly increased over the study period (p = 0.005). The GLp ‐1 group had higher median body mass indices (31.2, IQR: 7.4 vs. 29.3, IQR: 8.3; *p* = 0.024). Rates of peripheral arterial disease (68.8%), neuropathy (67.2%), and chronic kidney disease (18.2%) were similar between groups. Median A1c was similar between the GLp ‐1 group and controls (7.1, IQR: 2.4 vs. 7.4, IQR: 3.4; *p* = 0.333). Incidence of immediate flap success (100.0% vs. 96.6%, *p* =1.000), flap revision within the first 7 postoperative days (13.0% vs. 5.1%, *p* = 0.150), and partial flap necrosis (13.0% vs. 5.6%, *p* = 0.121) was similar between GLp ‐1 and control groups. By a median follow‐up of 16.8 (IQR: 27.2) months, rate of major lower extremity amputation trended to be lower in the GLp ‐1 group (4.4% vs. 21.1%, *p* = 0.086).

**Conclusions:** These preliminary data suggest that GLp ‐1 medication may have a positive benefit on long‐term limb salvage outcomes among diabetic patients with chronic LE wounds. With the continued rise in GLp ‐1 usage, further prospective investigation is warranted.

## 44 141

### 44.1 Diabetic Neuropathy Relief Using Topical Capsaicin or Topical Ketamine: A Systematic Review

#### 44.1.1 Kalyn Arnold; De'sia Blackwell, MS; Netanya Flores, MS; Sahil Kapadia; March Nembhard

44.1.1.1

**Background:** Diabetic peripheral neuropathy (DPN) is symptomatic of a lesioned nervous system and is characteristic of a loss of sensory function beginning in the lower extremities 1. Patients will experience burning at their feet, allodynia, hyperalgesia, numbness, and tingling. As an analgesic and dissociative anesthetic, Ketamine is a derivative of phencyclidine (PCP) and works to disrupt glutamatergic neurotransmission. It leads to a dissociative state and interacts with various other systems such as cholinergic, opioid, and monoaminergic pathways. Capsaicin is an exogenous agonist for the TRPV1 receptor on nociceptive nerve fibers and inhibits the pain initiation pathway. The aim of this study is to compare the current literature to determine the efficacy and preference of topical capsaicin versus topical ketamine when treating diabetic neuropathy.

**Methods:** A systematic review was performed using Central, PubMed, and MEDLINE, following PRISMA guidelines. The following terms were used in each database: topical capsaicin, topical ketamine, diabetic neuropathy, and peripheral neuropathy. Clinical studies were eligible and analyzed if it contained functional outcomes, complications, and visual analog scale (VAS) scores. The effect of topical ketamine and topical capsaicin improvement was based on overall reports of patient relief before the start of either treatment administration.

**Results:** Due to the heterogeneity of treatment protocols, more comparative trials that focus on true standardized dosages, treatment duration, and co‐administration with other medications are needed. 13 studies met the criteria for this systematic review out of the 52 studies initially identified. Topical capsaicin 0.075% applied three to four times a day for diabetic neuropathy showed reductions in pain intensity. Topical ketamine 5% in 1 mL dose also showed a reduction in symptomatic pain related to diabetic neuropathy. These results indicate that there is a greater reduction in pain intensity with the use of topical capsaicin but also related discomfort from possible burns. Further studies are needed for the long‐term efficacy of topical ketamine and topical capsaicin treatment on DPN.

**Conclusions:** While most ketamine and capsaicin administered treatments are effective for short‐term relief of neuropathic pain, additional investigations need to be inquired regarding patient inter‐individual variation and route of administration. Differences in doses between both topical and intravenous ketamine treatments, durations and adjunct medications can affect the length of immediate relief post‐treatment therapy. Standardizing treatment protocol with larger patient sample sizes would allow for cost‐effectiveness and increased patient satisfaction.

## 45 142

### 45.1 Metformin Improves Outcome in Digit Amputation

#### 45.1.1 Alyssa Miyasato, DPM; Hau Pham, DPM; Daniel Roh, MD, MPH; Subin Siby, DPM

45.1.1.1

**Background:** Chronic wounds are a common comorbidity associated with type II diabetes and metabolic dysfunction. Digit amputations are common in Diabetic foot ulcers that fail conservative treatment. Metformin is often used for diabetic patients. Studies showed it had senolytic properties, meaning it blocks senescent cells from secreting the senescence‐associated secretory phenotype, a combination of secretory molecules promoting chronic inflammation and tissue dysfunction. We hypothesized that patients using Metformin have better outcomes than those not using it when having digit amputation.

**Methods:** We reviewed retrospectively 259 patients who had digit amputation from 2013 to 2017. The Institutional Review Board approved an exemption for this review. Forty‐six patients used Metformin (group A). We matched with 46 patients not taking Metformin for age, gender, and year of surgery (group B). We compared the two groups' healing time and the need for more proximal amputations.

**Results:** In group A, 37 (80.4%) healed the digit amputation in 45 ± 26 days of patients who took Metformin. In group B, 28 (60.9%) of patients not taking Metformin healed in 47 ± 47 days. Group A had six (13%) midfoot amputations, and group B had nine (19.6%) (*p* = 0.1575). Group A had three (6.5%) major amputations, and group B had nine (19.6%) (*p* = 0.0461).

**Conclusions:** Metformin appears to improve healing in digit amputation. This study only reviewed surgical outcomes; a review with larger participants is needed for the conservative treatment of Diabetic foot ulcers using senolytic drugs versus other diabetic drugs.

## 46 143

### 46.1 Treatment Strategies for Closure of Posterior and Plantar Heel Ulcers: A Retrospective Comparative Analysis

#### 46.1.1 Danny Chamaa, MS; Sami Ferdousian, MS; Ryan Lin, MD; Rachel Rohrich

46.1.1.1

**Background:** Plantar heel ulcers result from improper biomechanics and neuropathy, while posterior heel ulcers occur due to pressure. This differentiation is overlooked in the literature. This study aims to compare the surgical treatment of posterior and plantar heel ulcers

**Methods:** A review of heel ulcers treated via local flap, free tissue transfer (FTT), and calcanectomy with primary closure from January 2014 to December 2019 was conducted.

**Results:** Of 113 heel ulcers, 56.5% (n=64) were posterior and 43.4% (n=49) were plantar. Patients with posterior heel ulcers were significantly older (64.9 ± 12.2 vs. 60.2 ± 11.5 years, *p* = 0.038). Other demographics were similar between groups. Posterior ulcers were more often treated with calcanectomy and primary closure (67.2% vs. 38.8%, *p* = 0.010), while plantar heel ulcers were more frequently treated with local flap or FTT. Median follow‐up time was 16 (IQR: 32.5) months: 67.3% experienced complications and 37.2% experienced postoperative infection. There were 28 cases of below‐knee amputation (posterior: 21.9% vs. plantar: 28.6%, *p* = 0.414). A significantly higher proportion of posterior ulcer patients were non‐ambulatory at final follow‐up (33.3% vs. 8.9%, *p* = 0.011). Mortality rate did not differ between groups (posterior: 21.9% vs. plantar: 28.6%, *p* = 0.511). Calcanectomy with primary closure resulted in the highest rates of any complication (p < 0.001), infection (p < 0.001), and postoperative below‐knee amputation (p = 0.023).

**Conclusions:** Posterior heel ulcer treatment should focus on preventing the formation of new pressure points. Treatment of the plantar heel should optimize a functional walking surface and correct biomechanics. Local flaps and FTT may be beneficial for both ulcer locations.

## 47 144

### 47.1 A Two Stage Surgical Approach for Successful Charcot Neuroarthropathy Limb Salvage

#### 47.1.1 Coleman Clougherty, DPM; Eric Hancock, DPM

47.1.1.1

**Background:** Charcot neuroarthropathy of the lower extremity has a significant impact on the

morbidity and mortality of patients and become more drastic for patients that have additional

comorbidities such as congestive heart failure, end stage renal disease, and diabetes mellitus.

When conservative options to offload and provide a functional limb fail, surgical intervention

becomes a final option prior to obtaining a major level amputation thus becoming a limb salvage scenario. Serial osteotomies and debridements can limit ulcerations and prevent infection in the short term, but numerous operations and undergoing physiological stress from anesthesia can cause significant harm to patients. The purpose of our study is to showcase successful Charcot reconstruction surgery in a safe and efficient way.

**Methods:** One of the techniques utilized at our institution incorporates a two‐stage surgical procedure protocol for Charcot neuroarthropathy patients who wish to pursue limb salvage versus a major amputation (i.e., below or above knee amputation). Our primary procedure includes correction of the underlying deformities but with soft issue and bone procedures, joint prep of the offending deformities, removal of ulcerations, then the application of an external fixation device to offload the plantar foot and stabilize patient's osseous integrity if they are in the destructive or coalescent phase of their pathology. After 6‐8 weeks of routine clinic visits, serial radiographs, and proper dressing care, the secondary stage takes place with removal of external fixation and incorporating stable internal fixation for a functional, ulcer free limb.

**Results:** In this study, a total of 35 Charcot reconstructive surgeries took place with the above

protocol with a single surgeon and one institution. 32 patients went on to heal and have a functional limb at a minimum one year post‐operative period from their initial procedure (91.4% salvage).

**Conclusions:** This study showcases the success of our two‐stage procedure in allowing patients to avoid major amputation in severe Charcot neuroarthropathy cases. Serial casting, debridements, and surgical interventions can prove to be harmful and costly to patients with Charcot deformities. Our approach has demonstrated great success in allowing patients to have minimal time in the operating room, quicker time to return to ambulation, and allowing patients to avoid life altering amputation surgeries.

## 48 145

### 48.1 LIMB‐Q Analysis of Prosthetic Function and Satisfaction After Below‐Knee Amputation

#### 48.1.1 Christopher Attinger, MD; Leila Jones; Ryan Lin, MD; Rachel Rohrich; Finn Tobias

48.1.1.1

**Background:** Achieving optimal prosthetic function is important for restoring mobility and quality of life following below‐knee amputation (BKA). However, not all patients achieve effective prosthetic use, and satisfaction with prosthesis fit, comfort, and appearance remains highly variable. The LIMB‐Q Prosthesis Modules, a validated patient‐reported outcome (PRO) measure, provides a standardized assessment of prosthetic function and prosthetic satisfaction. This study evaluates clinical factors associated with prosthetic function and satisfaction in a cohort of unilateral nontraumatic BKA patients using the LIMB‐Q.

**Methods:** A cross‐sectional survey study was conducted on patients with unilateral nontraumatic BKA presenting for routine follow‐up at a high‐volume limb center. Eligible patients had undergone BKA or lower extremity (LE) surgery at least six months prior, had no open wounds at the time of evaluation, and were using a prosthesis. The LIMB‐Q Prosthesis Module was administered during clinic visits to assess two key domains: Prosthesis: Function and Prosthesis: Satisfaction. The Prosthesis: Function scale (12 items) evaluates the extent to which patients can effectively use their prosthesis for physical activities, ambulation across different surfaces, and mobility in various settings. The Prosthesis: Satisfaction scale (12 items) measures patients’ satisfaction with the appearance, comfort, and fit of their prosthesis. Higher scores in both domains indicate better functional and subjective outcomes. Univariate regression analysis was performed to identify patient characteristics associated with prosthetic function and satisfaction.

**Results:** A total of 13 unilateral BKA patients were included. The cohort was predominantly male (76.9%) with a mean age of 65.6 ± 8.0 years and a mean BMI of 33.5 ± 5.6 kg/m2. Diabetes (92.3%), peripheral vascular disease (100%), neuropathy (76.9%), chronic kidney disease (61.5%), and end‐stage renal disease (15.4%) were prevalent. Most patients were ambulatory, with 46.2% walking independently and 46.2% requiring an assistive device. Higher BMI was significantly associated with worse prosthetic function scores (ß = ‐2.5, *p* = 0.032). Neuropathy was negatively correlated with prosthetic function, though it did not reach statistical significance (ß = ‐26.3, *p* = 0.075). No clinical variables were significantly associated with prosthetic satisfaction.

**Conclusions:** In this pilot study of 13 patients, higher BMI and neuropathy were associated with worse prosthetic function, likely due to mechanical and neuromuscular impairments affecting ambulation and mobility across different terrains. However, prosthetic satisfaction was not significantly influenced by medical comorbidities, suggesting that subjective perception of comfort, fit, and appearance may be shaped by factors beyond clinical status, including prosthetic customization, rehabilitation engagement, and psychological adaptation.

## 49 146

### 49.1 The Importance of Vigilant Vascular Surgery Surveillance in the Comorbid Lower Extremity Free Flap Population Undergoing Limb Salvage Surgery

#### 49.1.1 Sabrina DeLeonibus; Sami Ferdousian, MS; Ryan Lin, MD; Rachel Rohrich; Kishan Shah

49.1.1.1

**Background:** The current literature acknowledges the importance of vascular intervention before LE FTT, yet there is a lack of discussion regarding postoperative management. Thus, this study aims to characterize the population requiring postoperative revascularization after LE FTT, demonstrate the importance of long‐term follow‐up in this population, and guide efforts to enhance surveillance in this high‐risk group.

**Methods:** A retrospective review of LE FTT performed by a single surgeon at a single institution from was conducted.

**Results:** 356 LE FTT were performed with an immediate flap success rate of 96.1% and limb salvage rate of 87.1% over a follow‐up period of 25.7 ± 27.2 months. On univariate analysis, predictors of postoperative vascular intervention included diabetes (OR: 26.9, *p* = 0.001), higher charlson comorbidity indices (OR: 1.4, *p* < 0.001), peripheral vascular disease (OR: 7.3, *p* < 0.001), neuropathy (OR: 5.3, *p* < 0.001), chronic kidney disease (OR: 3.3, *p* = 0.008), the need for a preoperative endovascular intervention (OR: 17.1, *p* < 0.001), having an intraoperative thrombosis (OR: 18, *p* = 0.002) or revision (OR: 4.2, *p* = 0.005), calcified vessels (OR: 6.6, *p* < 0.001), partial flap necrosis (OR: 7.0, *p* = 0.001), infection (OR: 3.2, *p* = 0.008), recurrent ulceration (OR: 4.4, *p* < 0.001), and new ulceration (OR: 3.2, *p* = 0.003). Upon multivariate analysis, the only factor to remain significantly predictive of postoperative vascular intervention was preoperative vascular intervention (OR: 8.7, CI: 3.2‐23.6, *p* < 0.001).

**Conclusions:** Patients with extensive comorbidities affecting LE vasculature and that require preoperative vascular optimized before LE FTT must be monitored closely by a team of both vascular and plastic surgery specialists. However, in taking a vasculo‐plastic approach with long‐term surveillance, durable limb salvage is possible in this population despite higher rates of flap complications.

## 50 147

### 50.1 Use Of Circular Frame Fixation For Offloading In Lower Extremity Reconstruction: A Case Series

#### 50.1.1 Lawrence Colen, MD, FACS; William Grant, DPM; John Mancoll, MD; Ryan Mancoll

50.1.1.1

**Background:** Lower extremity limb salvage offers unique surgical challenges: the dependent nature of the lower leg, vascular disease, and the need for offloading. In this study, we look at a single surgeon's experience utilizing circular ring multiplanar external fixation to help protect flaps in lower extremity reconstruction.

**Methods:** This retrospective series (*n* = 47) reviewed cases from patients who received circular ring multiplanar external fixation after lower extremity reconstructive surgery between January 2013 and August 2021.

**Results:** Notable demographic data include a mean age (min, max) of 62.4 (37,88), a mean(St Dev) BMI of 30.7 (6.19), and a mean (St Dev) comorbidity number of 3.15 (1.7). Outcomes were an ambulation rate of 97.8%, complication rate of 27.6%, with a partial flap loss and complete flap loss rate both of 2.1%. Obesity (BMI > 30) was found to be a predictor of complications (P = 0.0031). As well, Factors like addition of skin grafting onto non covered flaps for closure and wound number trended towards being a significant predictor of complications.

**Conclusions:** While smaller reports have demonstrated value, this is the largest plastic surgery series showing external fixation's merits in safety and early ambulation. Podiatric data supports this role in wound healing, but hasn’t addressed complex soft‐tissue reconstructions. Despite design limits, our data suggests low rates of serious complications, as well as preoperative considerations to avoid complications. However, more data is needed to identify more predictors of complications and further support this technique as the standard of care.

## 51 148

### 51.1 From a LEAPP of Faith to DEFINITE Care: An Eight‐Year Journey of Multi‐Disciplinary Care of Diabetic Foot Ulcers in Singapore

#### 51.1.1 Sadhana Chandrasekar, MD, FACS; Jaime Lin, MD, MSc, MBA; Zhiwen Lo, MD, FACS

51.1.1.1

**Background:** Singapore has a high burden of diabetic foot ulcers with the highest major lower extremity amputation (LEA) rates amongst OECD countries. Multi‐disciplinary team (MDT) management of DFU has been shown to have a significant impact on outcomes and reduces the risk of major amputations.

**Methods:** We recount our journey on the path to better outcomes, starting with the establishment of the first MDT foot clinic (Lower Extremity Amputation Prevention Programme) in 2017. Subsequently, with the help of a population health grant, the DEFINITE (Diabetic Foot in Primary and Tertiary) Care programme was commenced in 2020. DEFINITE care is a health services innovation which enabled primary care patients with DFU rapid access to MDT LEAPP clinics at tertiary hospitals, with care coordination by Diabetic Foot Coordinators after diabetic limb salvage. Cost effectiveness analysis and patient reported outcome measures were also studied over the three year period.

**Results:** LEAPP clinic reduced waiting times for first visit from 35 days to 9 days and showed a 30% reduction in major LEA rates as compared to the previous year. MDT care within DEFINITE was associated with highly significant reductions in the composite major LEA/mortality rate by 9%, inpatient episodes and length of stay, emergency department visits and better DM control. DFU healing was associated with improved EQ5D utility value and PROMs.

**Conclusions:** MDT care was cost effective with an ICER of S$30564 per QALY gained.

## 52 149

### 52.1 Quantifying Irregularity and Asymmetry in Gait Dynamics for Transmetatarsal Amputation Patients

#### 52.1.1 Nori Pham; Rachel Rohrich; Kishan Shah; Jie Jung Shih, MS; Finn Tobias

52.1.1.1

**Background:** Transmetatarsal amputations (TMA) preserve the ankle joint, making them preferable to more proximal amputations. However, research on gait dynamics in TMA patients is limited. This study aims to characterize gait after nontraumatic TMA in a chronic wound population in order to identify potential gait‐related factors that may influence comorbidities and enhance patient outcomes.

**Methods:** A single center, cross‐sectional study was conducted from June 2021 to September 2024. Participants completed a 120‐second walk test with wearable gait sensors. Demographic, medical and surgical histories were documented. A variety of gait parameters were measured and compared between the limb of TMA and limb of no surgery.

**Results:** 8 patients underwent TMA and completed gait testing. The analysis revealed a significant difference in stride length between the amputated leg and the non‐amputated leg (0.64 [0.15] vs 1.05 [0.40], *p* = 0.027)). Gait parameters such as speed and single limb support (35.20 ± 2.91 vs 34.94 ± 2.89, *p* = 0.859) showed no significant differences.

**Conclusions:** Gait symmetry was maintained in the TMA population, with balanced limb support. However, stride length varied due to the TMA limb's shorter foot, resulting in reduced push‐off force while walking. TMA effectively enhances functional gait outcomes and should be prioritized over more proximal amputations. Regular follow‐ups on patients’ lower extremity function is essential and focused efforts should be made to reduce the risk of falls or imbalance gait, and prevent further reductions in stride length.

## 53 150

### 53.1 Does Diabetes Type Influence Healing Outcomes after Lower Extremity Free Tissue Transfer? A Comparative Study

#### 53.1.1 Sami Ferdousian, MS; Winnie Li; Rachel Rohrich; Isabel Snee; Harrison Thomas, MD

53.1.1.1

**Background:** Diabetes mellitus (DM) is known to impact healing outcomes in lower extremity (LE) free tissue transfer (FTT), especially when accompanied by other comorbidities. Given their distinct pathophysiology, T1 and T2 may differentially affect healing pathways; however, this association is not well studied. Thus, our study aims to characterize healing differences in T1 and T2 diabetics undergoing LE FTT for chronic LE wounds.

**Methods:** A retrospective chart review of patients undergoing LE FTT from November 2016 to January 2023 was conducted. Demographics, medical history, vascular characteristics, and outcomes were collected. Patients were identified as either T1 or T2 diabetics and compared. Flap success was defined as flap viability within the first 7 postoperative days. Primary outcomes were complication rates, including dehiscence, infection, and revision operation, and flap success.

**Results:** Of 291 consecutive patients undergoing 300 LE FTT, 160 (55.0%) patients were diabetic and underwent a total of 164 LE FTT. Of these patients, 15 (9.4%) were T1 and 145 were T2 (90.6%). There were no significant differences in comorbidities or demographics, with the exception of age and BMI (Table 1). Overall, 29.9% required an endovascular intervention before FTT, with no differences between T1 and T2 groups (n=3, 18.8% vs. n=46, 31.1%; *p* = 0.397). The median wound size was 82.5 (IQR: 70), with no differences between groups (p = 0.848). Muscle flaps comprised 42.7% of cases, while fasciocutaneous flaps comprised 47.6%, with no differences in their use between groups (p = 0.659 and *p* = 0.464, respectively). Patients were followed for a median of 15.3 (IQR: 24.6) months. Flap success rate was similar between T1 and T2 groups (n=15, 93.8% vs. n=143, 96.6%; *p* = 0.465). However, T1 diabetics experienced significantly higher rates of any flap complication (n=10, 62.5% vs. n=50, 33.8%; *p* = 0.030) and the need for a revision operation after postoperative day 7 (n=3, 18.8% vs. n=4, 2.7%; *p* = 0.021) compared to the T2 group (Table 2). Rate of dehiscence, infection, and immediate flap takeback were similar between groups.

**Conclusions:** While flap success is comparable between T1 and T2 diabetics, T1 diabetics may be at higher risk for wound healing complications. Further study of this complex subpopulation is warranted.

## 54 151

### 54.1 Review of Lisfranc and Chopart Amputation

#### 54.1.1 Ashley Daniel, DPM, MPH; Alyssa Miyasato, DPM; Hau Pham, DPM; Wei Tseng, DPM

54.1.1.1

**Background:** A diabetic foot ulcer is a major complication of diabetes. Every year in the United States, there are 73,000 non‐traumatic leg amputations in people with diabetes, and an ulcer precedes 80% of these. Failed partial foot amputations lead to major amputations. Lisfranc (LFA) and Chopart amputations (CPA) are controversial but have been used to avert major amputations. We hypothesized that these amputations can help prevent or reduce major amputations.

**Methods:** We reviewed 64 participants who received care between 2014 and 2023. We excluded participants who didn’t have diabetes, died while healing, or were lost to follow‐up. Co‐morbidities include Chronic Kidney Disease, End‐Stage Renal Disease, Coronary Artery Disease, Congestive Heart Failure, and smoking. We evaluated the healing rate and the need for major amputations in 12 months. This study received an exemption from our Institution Review Board.

**Results:** Forty‐nine participants, 18 females, and 31 males met the inclusion criteria. The average age was 64 (37 to 88). There were 35 CPAs and 14 LFAs. Overall, 33 participants (67%) healed and preserved the limbs, 11 of whom had LFAs and 22 CPAs. The average time to recover was 132 days. At the 12‐month follow‐up, one participant had AKA and one BKA after healing the LFAs and CPAs.

**Conclusions:** Facing major amputation was a difficult task; our study showed that LFA and CPA were good alternatives. Our number is small, but the study showed that 2/3 of limbs that would have been lost were salvaged.

## 55 152

### 55.1 The Mental Cost of Limb Loss: Psychiatric Comorbidities Are Associated With Poorer Function After Below‐Knee Amputation, A LIMB‐Q Analysis

#### 55.1.1 Christopher Attinger, MD; Leila Jones; Ryan Lin, MD; Rachel Rohrich; Finn Tobias

55.1.1.1

**Background:** Psychiatric comorbidities are prevalent in patients with limb loss, yet their influence on functional recovery and quality of life remains underappreciated. The LIMB‐Q, a validated, patient‐reported outcome (PRO) measure for this population, provides comprehensive assessment across function, symptoms, psychological well‐being, financial burden, and prosthetic satisfaction. This study evaluates the association between psychiatric diagnoses and LIMB‐Q scores following nontraumatic BKA.

**Methods:** A cross‐sectional survey study was conducted on patients who underwent nontraumatic BKA and presented for routine follow‐up at a high‐volume limb center. Eligible patients had undergone BKA or lower extremity (LE) surgery at least six months prior and had no open wounds at the time of evaluation. The LIMB‐Q, a validated patient‐reported outcome measure, was administered during clinic visits to assess function, symptoms, psychological impact, financial burden, and work limitations. Higher scores reflected better outcomes in each domain. Univariate regression analysis was performed to identify associations between psychiatric comorbidities and LIMB‐Q outcomes.

**Results:** A total of 13 unilateral BKA patients were included. The cohort was predominantly male (76.9%) with a mean age of 65.6 ± 8.0 years and a mean BMI of 33.5 ± 5.6. Diabetes (92.3%), peripheral vascular disease (100%), neuropathy (76.9%), chronic kidney disease (61.5%), and end‐stage renal disease (15.4%) were prevalent. Nearly half of the cohort (46.2%) had a documented psychiatric diagnosis, with depression and anxiety each affecting 38.5%. Patients with a psychiatric diagnosis had significantly lower function scores (ß = ‐30.3, *p* = 0.014). Anxiety (ß = ‐32.6, *p* = 0.007) and depression (ß = ‐25.1, *p* = 0.058) were both associated with worse function. Depression was also significantly associated with greater BKA‐related symptom burden (ß = ‐22.7, *p* = 0.027). End‐stage renal disease was significantly associated a greater psychological impact of BKA (ß = ‐52.3, *p* = 0.011).

**Conclusions:** In this pilot study, psychiatric comorbidities were strongly associated with poorer function and greater symptom burden following BKA. While limited by a small sample size and a lack of preoperative assessment, these findings highlight the need to integrate psychiatric services within amputee rehabilitation to improve functional recovery, optimize prosthetic adaptation, and enhance overall quality of life.

## 56 153

### 56.1 Retrospective Analysis of Understudied Real‐world Complicated and Persistent Diabetic Foot Ulcers: Use of Retention‐Processed Placental Membranes Vs SOC

#### 56.1.1 Robert Frykberg, DPM, MPH; Toni‐Ann Martorano, MS; Zwelithini Tunyiswa; Wendy Weston, PhD

56.1.1.1

**Background:** Diabetic foot ulcers (DFUs) are a severe complication for diabetic patients and frequently leads to amputations. Standard of Care (SOC) treatments are often inadequate, necessitating advanced wound care products, such as placental allografts. We conducted a real‐world, retrospective analysis comparing the effectiveness of retention‐processed human amnion/chorion membrane (RE‐AC) versus SOC in chronic DFU treatment. The wounds studied exhibited very hard‐to‐heal properties and would generally be eliminated from clinical studies.

**Methods:** Retrospective data was collected from electronic health records of patients treated with RE‐AC at three wound care centers. Synthetic control SOC patients were matched from a wound registry using Coarsened Exact Matching. The analysis employed Bayesian regression and Hurdle Gamma Analysis of Variance (ANCOVA) models. Wounds measured on average 13.8 cm2, the largest being 20.8 cm2. Of the wounds in the study, 7 had osteomyelitis, 4 had infections, and 1 had undermining, and present for over 385 days prior to treatment.

**Results:** Results indicated that RE‐AC achieved approximately 10% higher Expected Wound Closure Rate compared to SOC at 12 weeks. For wounds that did not close, RE‐AC resulted in about 60% Expected Percent Area Reduction, whereas SOC wounds stalled or grew larger.

**Conclusions:** The findings suggest that RE‐AC is superior to SOC as a covering for wound treatment. This benefit likely leads to reduced treatment costs, improved outcomes in the DFU patient population, and subsequently less amputations.

## 57 154

### 57.1 The Psychofunctional Impact of Length Preservation in Various Levels of Pedal Amputation

#### 57.1.1 Jayson Atves, DPM; Alex Dang, DPM; Karen Evans, MD; Ali Qadri, DPM; Craig Verdin, DPM

57.1.1.1

**Background:** Diabetes‐related foot amputations are challenging not only due to medical comorbidities but also the psychological distress they cause, with diabetic patients being twice as likely to experience depression compared to the general population. These amputations often exacerbate psychological issues, including heightened depression and anxiety. Our focus is to highlight the functional and psychological benefits of length preservation in diabetic foot amputations, which can improve both mobility and emotional well‐being.

Diabetes‐related foot amputations are particularly challenging for patients, not only due to medical issues but also psychological distress. About 25% of individuals with diabetes will experience a diabetic foot wound, significantly increasing the risk of amputation, elevate depression and anxiety levels (1,2,3). Pedras et al. found that pre‐surgery anxiety and depression predicted similar issues a month post‐amputation. Anxiety was notably higher than depression before surgery but decreased afterward, with both contributing to post‐surgery depression. Additionally, amputations can significantly alter self‐identity, influenced by personal reactions, rehabilitation goals, and social support (4).

A study by Sinha et al. showed that amputees had significantly lower quality of life scores compared to non‐amputees, with factors like employment status, prosthetic use, comorbidities, and pain affecting overall well‐being. These factors highlight the importance of addressing the needs of amputees to improve reintegration and quality of life (5). Amputees often face unmet needs for information on financial assistance, employment, and social activities, leading to social isolation and psychiatric issues. Well‐fitting, inconspicuous prosthetics can boost confidence and support rehabilitation, while more visible devices may contribute to social anxiety and body image concerns (6,7,8,9,10).

This study focuses on the benefits of length preservation in diabetic foot amputations, aiming to improve both psychological and functional outcomes for patients by enhancing mobility and self‐image.

**Methods:** This comparative retrospective analysis include 65 unilateral ambulatory midfoot amputees (8 pandigital, 43 transmetatarsal, 5 Lisfranc, and 9 Chopart) who were evaluated at Georgetown University Hospital between June 2021 to June 2023. Outcomes were defined as complications (major and minor), rates of limb salvage, assessment of residual function, psychological well‐being, and quality of life using validated patient reported outcome measures including the Lower Extremity Functional Scale (LEFS) and the SRQ‐20. The LEFS scale consists of 20 items that patients rate based on their ability to perform specific activities scored from 0 (extreme difficulty or inability to perform the activity) to 4 (no difficulty).

**Results:** This study of 65 patients (mean age 64.6 years) undergoing various foot amputations found a high limb salvage rate (96.36%) but a notable complication rate (21.8%). Follow‐up duration averaged 12.1 months. Diabetes was prevalent in 50 patients, and comorbidities like chronic kidney disease and peripheral vascular disease were common. Patient‐reported outcomes indicated significant differences in quality of life, with the Chopart group reporting the lowest SF‐12 scores (25.5), while the Pandigital group had the highest (31.8). The Lisfranc group had the highest incidence of major complications (p = 0.02), and 22 patients required additional surgery.

**Conclusions:** Our study highlights the significant outcomes associated with midfoot amputations, particularly the high limb salvage rate of 96.36% and an overall complication rate of 21.8%. While the Lisfranc group exhibited a notably higher incidence of major complications, minor complications were similar across groups, suggesting that while the type of amputation may influence the severity of complications, minor issues may be more uniformly managed across different amputation types.

Pandigital amputations showed an advantage over other forms of pedal amputation in terms of functionality and psychological well‐being. It was noted that length preservation is associated with reduced psychological burden and a lesser impact on quality of life compared to shorter residual limbs. The statistical significance observed in the SF‐12 (p = 0.01) highlights the critical role of mental health in recovery, suggesting that interventions aimed at improving psychological outcomes should be a focal point in rehabilitation programs. These findings underscore the importance of individualized treatment strategies that consider both the physical and psychological aspects of amputation, ultimately enhancing patient outcomes and overall quality of life. To our knowledge, this study is the first to evaluate the psychofunctional impact of all levels of midfoot amputation, a common intervention utilized in limb salvage. Future research is needed to further investigate the long‐term effects of various amputation levels on patient outcomes, particularly focusing on larger sample sizes and diverse populations.

## 58 155

### 58.1 Neck Circumference As A Simple Tool For Identifying Patients At Risk For Skin Graft Failure

#### 58.1.1 Rohan Thakur, MD

58.1.1.1

**Background:** Neck circumference (NC) is an accessible measure of adiposity in addition to being a clinical marker for cardiovascular disease, metabolic syndrome, obstructive sleep apnea, and insulin resistance. Consequently, NC may be a marker for poor wound healing and impact surgical outcomes. Our study aims to determine if NC impacts split‐thickness skin graft (STSG) outcomes in patients with chronic lower extremity (LE) wounds.

**Methods:** A review of patients receiving STSG for atraumatic LE wounds between 2014 and 2022 was conducted. Patients with a NC = 42cm (NC‐42) were compared to patients with a NC < 42cm. Primary outcome was graft failure.

**Results:** A total of 145 patients with 211 wounds underwent STSG: 62 (42.8%) were NC‐42 and 83 (57.2%) were not. The median age was 61 (IQR: 22) years with a Charlson Comorbidity Index of 5 (IQR: 3). The NC‐42 group demonstrated a higher BMI (32.8 vs. 26.2, *p* < 0.001), and prevalence of males (72.6% vs. 49.4%, *p* = 0.005), diabetes (67.7% vs. 44.6%, *p* = 0.006), and hypertension (93.6% vs. 69.9%). Other demographic and wound characteristics were similar between groups. Graft failure was significantly higher in the NC‐42 group (22.3% vs. 10.3%, *p* = 0.016). A multivariate regression accounting for co‐variates to failure demonstrated NC‐42 as independently predictive of graft failure (OR: 2.4, *p* = 0.031).

**Conclusions:** A NC = 42 independently predicts graft failure in highly comorbid patients with chronic LE wounds. NC may be a useful clinical marker to identify high‐risk patients and plastic surgeons should work closely with medicine to optimize these patients’ healing perioperatively.

## 59 156

### 59.1 Quantifying Gait Symmetry in Patients Following Lower Extremity Free Tissue Transfer: A Cross‐Sectional Study

#### 59.1.1 Meghan Currin; Karen Evans, MD; Leila Jones; Ji Jung Shih, MS; Nori Pham

59.1.1.1

**Background:** Recovery of a functional gait with minimal imbalance is a primary post‐operative goal of free tissue transfer (FTT) in diabetic patients who tend to have chronic, non‐traumatic lower extremity (LE) wounds. However, no literature quantifies gait symmetry changes following LE FTT. Thus, this study aims to assess interlimb spatiotemporal gait parameters in patients who have undergone LE FTT.

**Methods:** A single center, cross‐sectional study was conducted from June 2021 to September 2024. Patients who underwent unilateral FTT without partial foot amputation completed a 120‐second walk test. Gait parameters from FTT limb and non‐FTT limb were compared.

**Results:** A total of 11 patients were included. The average age was 58.0 ± 12.8 years, median BMI was 31.3 (IQR: 3.33), and the average Charlson Comorbidity Index was 4.7 ± 2.3. Stride length was significantly longer when leading with the FTT limb (0.82 ± 0.42) compared to the non‐FTT limb (0.70 ± 0.26, *p* = 0.029). There were no significant differences in other gait parameters, including gait speed (0.81 ± 0.38 vs 0.85 ± 0.41, *p* = 0.192), elevation mid‐swing (1.89 ± 1.42 vs 1.74 ± 2.30, *p* = 0.929), step duration (0.60 ± 0.09 vs 0.64 ± 0.09, *p* = 0.067), and cadence (100.63 ± 12.50 vs 101.12 ± 05.01, *p* = 0.929).

**Conclusions:** Minimal gait differences were observed between the FTT limb and the non‐FTT limb, except for a significant increase in stride length in the FTT limb, suggesting FTT may help maintain functional symmetry during ambulation.

## 60 157

### 60.1 The Learning Curve in Free Tissue Transfer for Limb Salvage in an Increasingly Complex Population

#### 60.1.1 Meghan Currin; Karen Evans, MD; Christopher Attinger, MD; Kenneth Fan, MD; Sami Ferdousian, MS; Ryan Lin, MD; Rachel Rohrich; Richard C. Youn, MD

60.1.1.1

**Background:** The process of becoming skilled at a technique can be referred to as the learning curve (LC). This study characterizes the LC in microsurgical free tissue transfer (FTT) in an increasingly complex limb savage population.

**Methods:** Lower extremity (LE) FTT were reviewed from July 2011 to October 2024. Operative durations (OD) were utilized to define the LC. The number of procedures at which OD stabilized was used to define competency phases.

**Results:** 356 LE FTT were performed with a median OD of 402 (IQR: 161.5) minutes. Each additional month since study initiation was associated with a significant decrease of 1.1 min in OD (CI: ‐1.3 to ‐0.8, *p* < 0.001). OD stabilization occurred after 203 FTT, creating two distinct phases: learner phase (1‐203 cases) and mastery phase (final 153 cases). Median age was significantly higher in the mastery phase (4, IQR: 3, vs. 4, IQR: 4; *p* = 0.044). The mastery phase also had a significantly higher proportion of patients with diabetes (65.4% vs. 48.3%, *p* = 0.001) and peripheral arterial disease (61.4% vs. 48.8%, *p* = 0.018). The requirement of intraoperative transfusion trended to be lower in the mastery phase (28.7% vs. 37.9%, *p* = 0.078). Patients in the mastery phase experienced a significantly shorter time to functional ambulation (64, IQR: 59 vs. 124.5, IQR: 280.5 days; *p* < 0.001). Rates of takeback for flap revision, flap success, and major limb amputation did not differ by phase.

**Conclusions:** Increased surgical proficiency in lLE FTT enabled limb salvage in patients who may not have been candidates earlier in the learning curve due to comorbid constraints. These findings highlight the role of high‐volume limb salvage centers in continuing to expand reconstructive options for this challenging population, offering life‐ and limb‐saving interventions to patients with severe comorbidities who might otherwise face amputation.

## 61 158

### 61.1 Assessing the Predictive Value of the Charlson Comorbidity Index in Lower Extremity Free Tissue Transfer for Limb Salvage: A 13‐Year Retrospective Cohort Study

#### 61.1.1 Meghan Currin; Karen Evans, MD; Sami Ferdousian, MS; Ryan Lin, MD; Rachel Rohrich

61.1.1.1

**Background:** The Charlson Comorbidity Index (CCI), a weighted index that assesses a patient's risk of death based on their comorbidities, has shown value in predicting adverse events. This study investigates the utility of the CCI in the complex limb salvage population receiving lower extremity (LE) free tissue transfer (FTT).

**Methods:** A retrospective review of LE FTT from July 2011 to October 2024 was conducted. Prolonged postoperative length of stay (LOS) was defined as greater than 75th percentile of the cohort. Major amputation was defined as below‐knee and other proximal amputations after LE FTT.

**Results:** A total of 356 LE FTT were performed in 346 patients. The median CCI was 4 (IQR: 3). Average hospital LOS and postoperative LOS were 26.5 (IQR: 16) and 14 (IQR: 8.5) days, respectively. Prolonged postoperative LOS occurred in 89 patients (25.0%), flap complications in 103 (28.9%), and amputation in 46 (12.9%) during the follow‐up period of 15.7 (IQR: 27.5) months. Mortality rate was 5.8% (n=20). Univariate analysis demonstrated that higher CCI was significantly associated with prolonged LOS, flap complications, amputation, and mortality. Upon multivariate analysis adjusting for significant co‐variates, CCI was not independently predictive of any primary outcome.

**Conclusions:** This is the largest cohort study evaluating the utility of CCI in patients undergoing LE FTT for limb salvage. While a higher CCI may correlate with adverse outcomes, it is not an independent predictor when adjusting for other factors.

## 62 159

### 62.1 Predictive Factors of Wound Bed Sterility After Serial Debridement Among a Large Cohort of Patients Undergoing Microsurgical Reconstruction for Limb Salvage

#### 62.1.1 Joshua Carreras; Sami Ferdousian, MS; Ryan Lin, MD; Rachel Rohrich; Sahil Sharma

62.1.1.1

**Background:** Chronic lower extremity (LE) wounds are serially debrided to achieve a sterile wound bed free of pathogens. This study aims to evaluate the wound characteristics that influence the microbial burden and their potential impact on the success of LE FTT for limb salvage.

**Methods:** A retrospective review of patients undergoing LE FTT was conducted. Data on the number of in‐house debridements and qualitative culture results from the first and final debridements were recorded.

**Results:** 193 patients were identified who underwent LE FTT and had culture data available from the date of surgery. The median age was 57 (IQR: 65‐48) years and mean BMI was 29.7 ± 6.0 kg/m2. Univariate linear regression analysis identified several factors associated with increased odds of microbial burden at FTT inset. These included positive initial debridement cultures (OR: 2.0, *p* = 0.038), longer hospital length of stay (OR: 1.0, *p* < 0.001), diabetes (OR: 3.33, *p* < 0.001), and higher values of ESR (OR: 1.1, *p* = 0.019), CRP (OR: 1.0, *p* = 0.002), and hemoglobin a1c (OR: 1.11, *p* = 0.025). The presence of indwelling hardware predicted wound bed sterility (OR: 2.3, *p* = 0.031). Wound area or the number of preoperative debridements did not impact odds of wound bed sterility. Upon multivariate analysis, higher preoperative CRP remained significantly associated with slightly increased odds of microbial burden in the wound bed (OR: 1.02, *p* = 0.011).

**Conclusions:** Diabetes, initial positive cultures, and high inflammatory markers predicted persistent bacterial burden, while wound size and number of debridements did not significantly correlate with wound bed sterility.

## 63 160

### 63.1 Post‐Procedural Management After Deep Venous Arterialization

#### 63.1.1 William Brownell, DPM; Brett Chatman, DPM; Elizabeth Genovese, MD, FACS; Allysa Vavra, DPM, MS

63.1.1.1

**Background:** In the United States, approximately 185,000 individuals undergo major amputations annually, with vascular disease and diabetes being the primary causes. Critical Limb‐Threatening Ischemia (CLTI) carries a grave prognosis, with reported mortality rates of 20% at six months and 25%–30% at one year (1). For patients undergoing major amputation, the one‐year mortality ranges from 13%–40%, increasing to 35%–65% at three years and 39%–80% at five years (4).

Transcatheter arterialization of the deep veins (TADV) is an innovative procedure designed as a last‐resort intervention for “no‐option” CLTI patients. This minimally invasive technique creates an artery‐to‐vein connection near the diseased tibial artery, rerouting oxygenated blood into the tibial veins to perfuse the soft tissues of the foot, offering new hope for limb salvage.

**Methods:** This study investigates podiatric post‐procedural management following deep venous arterialization (DVA), specifically examining the critical factors of amputation timing, surgical techniques, and wound care strategies in patients with Rutherford class 5 and 6 chronic limb‐threatening ischemia (CLTI). Our goal was to assess the effectiveness of a structured post‐procedural management plan, including surgical timing, techniques and wound care protocols, in effort to optimize wound healing and patient recovery for the “no‐option” patient population.

Our research centers on creating a podiatric surgical timeline and wound care management plan for patients requiring partial foot amputation after DVA. By addressing the clinical need for standardized guidelines in this state‐of‐the‐art vascular intervention, we strive to contribute evidence‐based recommendations that enhance recovery, promote wound healing, and support the advancement of multidisciplinary healthcare protocols.

**Results:** Patients attended bi‐weekly office visits with podiatric & vascular surgery for wound monitoring with continued local wound care, allowing time for neovascularization of the foot.

Staged partial foot amputation:

o Week 6‐8:

o Stage 1: Disarticulation of digit(s) at the metatarsophalangeal joint, with cartilage left intact

o Stage 2: (~48 h later ) Blunt retraction of tissue followed by minimal metatarsal resection, minimal closure over metatarsal resection with a skin substitute graft applied

o Stage 3: (~48 h later ) Negative pressure wound therapy (NPWT) applied over the wound/graft (bedside)

Postoperative Care:

o Bi‐weekly clinic visits for wound progress monitoring which included wound measurements, serial minimal debridement, additional skin graft applications when necessary & wound VAC changes qMWF by nursing

**Conclusions:** Time to healing was documented for all patients, with outcomes analyzed to assess success rates.

## 64 161

### 64.1 Synergistic Effects of Combination Negative Pressure Wound Therapy and Continuous Topical Oxygen Therapy in the Management of Ischemic Diabetic Foot Wounds

#### 64.1.1 Siti Rasyidah Binti Sampah, RN, BSN, CCRN, CWON; Yongsheng Chen, MBBS, DTMH; O Wern Low, MBBS, DTMH; Vigneswaran Nallahamby, MBBS, DTMH; Vikram Vijayan, MBBS, DTMH

64.1.1.1

**Background:** Negative pressure wound therapy

(NPWT) is widely used to improve tissue granulation and reduce wound healing times. However, in ischaemic wounds, it can lead to increased microvascular complication, thus worsening wounds and hence is contraindicated. We report our experience using a combination of NPWT and continuous topical oxygen therapy (CTOT) to heal ischaemic diabetic foot ulcers (DFU).

**Methods:** 4 patients who had no improvement in their DFUs despite revascularization, sepsis clearance and treatment with NPWT or CTOT individually, were chosen for combination therapy.

Sharp wound debridement was performed prior to applying CTOT

(NatroxTM) directly to the wound bed with oxygen delivery at 11mls/hr. Subsequently, standard NPWT (ActiVacTM) was applied at a sub‐atmospheric pressure of between 75 and 100mmHg, alternating between 2 min of suction and 10 min of rest, to ensure adequate delivery of oxygen via the CTOT system. Photo documentation and wound analysis was performed at weekly dressing changes which occurred either in the hospital or outpatient setting.

**Results:** All patients tolerated the novel therapy well. At baseline, all 4 patients had over 70% of their wound bed as slough and eschar. After 1 week of combination therapy, all patients had approximately 50% slough and increased areas of granulation on photo‐documentation analysis. 4 weeks later, all DFUs demonstrated over 50%

granulation tissue with no eschar observed and reduced wound size and depth.

**Conclusions:** This case series demonstrates that combined NPWT/CTOT

improves wound healing and has positive outcomes for complex, ischemic DFUs which warrants further investigation with a larger trial.

## 65 162

### 65.1 The Prognostic Impact of Medial Arterial Calcification in Primary Atraumatic Below‐Knee Amputation: A Large Retrospective Cohort Study

#### 65.1.1 Sami Alahmadi; Christopher Attinger, MD; Karen Evans, MD; Ryan Lin, MD; Amy Zhou

65.1.1.1

**Background:** Medial arterial calcification (MAC) is a marker of advanced vascular disease commonly seen in diabetes, end‐stage renal disease (ESRD), and peripheral arterial disease (PAD). Prior studies link MAC to poor limb salvage, but its impact on outcomes following primary below‐knee amputation (BKA) for non‐healing wounds remains unclear. This study investigates the prognostic significance of MAC in BKA patients.

**Methods:** A retrospective cohort study included patients undergoing primary BKA at a tertiary care wound center from September 2017 to January 2022. Pedal radiographs were scored by two reviewers using the Ferrassi et al. MAC grading scale. Patients were categorized into absent MAC (0) and any MAC (1‐5) groups.

**Results:** Among 259 patients, 152 (58.7%) had no MAC, and 107 (41.3%) had MAC. MAC patients had higher rates of diabetes, ESRD, and PAD (*p* < 0.001). All patients were ambulatory at baseline. Postoperative ambulation rates were similar between MAC and no MAC groups (59.6% vs. 64.5%, *p* = 0.432). Dehiscence occurred more frequently in the MAC group (18.7% vs. 8.6%, *p* = 0.016). Infection, hematoma, and revision rates were similar between groups (*p* > 0.08). Conversion to above knee amputation rates were similar between MAC and no MAC groups (0.9% vs. 0.7%, *p* = 0.657). Two‐year mortality was higher in MAC patients (19.6% vs. 10.5%, *p* = 0.039). Multivariate regression identified Charlson Comorbidity Index as an independent mortality predictor (*p* < 0.001), but not MAC (*p* < 0.2).

**Conclusions:** MAC is associated with higher dehiscence and 2‐year mortality following BKA but not overall mortality. Preoperative optimization may improve outcomes in this high‐risk population.

## 66 163

### 66.1 The Critical Impact of Limb Length Preservation: Patient‐Reported Outcomes Favor Below‐ Knee Over Above‐Knee Amputations

#### 66.1.1 Christopher Attinger, MD; Danny Chamaa; Ryan Lin, MD; Rachel Rohrich; Isabel Snee

66.1.1.1

**Background:** Below‐knee amputation (BKA) and above‐knee amputation (AKA) are last‐resort treatments for chronic lower extremity (LE) wounds. Quality‐of‐life data comparing AKA and BKA are limited. This study is the first to compare patient‐reported outcome measures (PROMs) between BKA and AKA patients.

**Methods:** PROMs were collected from patients undergoing BKA or AKA at a tertiary wound center between June 2022 and October 2024. Mental well‐being (SRQ‐20), pain intensity (PROMIS‐3a), resilience (CD‐RISC), and function (LEFS) were measured at 1, 3, and 6 months and 1, 2, and 5 years postoperatively.

**Results:** Of 96 patients, 22 (22.9%) underwent AKA and 74 (77.1%) underwent BKA. Mean age was 60.4 ± 13.6 years. Median survey completion time points were similar (*p* = 0.845). AKA patients had lower function (LEFS; 33.9 ± 13.7 vs. 47.4 ± 25.2; *p* = 0.003), higher psychological distress (SRQ‐20; 5.6 ± 4.3 vs. 3.1 ± 3.9; *p* = 0.002), and greater pain intensity (PROMIS‐3a; 58.9 ± 10.4 vs. 48.2 ± 13; *p* = 0.001). Resilience scores were comparable (CD‐RISC; 7 ± 1.2 vs. 7 ± 1.4; *p* = 0.964).

**Conclusions:** This is the first study comparing PROMs between AKA and BKA patients. AKA patients experience more pain, psychological distress, and lower function, suggesting a greater need for targeted postoperative support and preserving the knee joint when possible. The relative heterogeneity of PROMs across groups may reflect that the level of amputation decision was made for the intended patients.

## 67 164

### 67.1 Mental Health in Chronic Lower Extremity Wound Care: An Analysis of Psychological Distress, Pain, and Functional Outcomes

#### 67.1.1 Christopher Attinger, MD; Danny Chamaa; Sami Ferdousian, MS; Ryan Lin, MD; Rachel Rohrich

67.1.1.1

**Background:** Chronic lower extremity (LE) wounds cause pain, reduced mobility, and psychological distress, impacting quality of life. Common mental disorders (CMD) may be prevalent in this population, but systematic screening is uncommon. This study examines the impact of CMD on patient‐reported outcomes in chronic LE wound patients.

**Methods:** A cross‐sectional survey of patients with chronic LE wounds treated at a multidisciplinary clinic from June 2022 to December 2024 was conducted. Data included demographics, comorbidities, psychiatric diagnoses, and wound care history. Validated questionnaires assessed mental well‐being (SRQ‐20), quality of life (SF‐12), pain intensity (PROMIS‐3a), resilience (CD‐RISC), and function (LEFS).

**Results:** Among 714 patients, 108 (15.1%) had psychiatric diagnoses, mainly depression or anxiety. Wound type and surgical history did not differ significantly (*p* > 0.21). CMD patients had higher SRQ‐20 scores (5.3 vs. 3.6; *p* < 0.0001), greater suicidal ideation (8.4% vs. 3.4%; *p* = 0.023), more intense pain (PROMIS‐3a: 56.2 vs. 52.7; *p* = 0.016), and lower functional abilities (LEFS: 42.8 vs. 48.3; *p* = 0.04). CMD patients were more likely to seek mental health care (*p* = 0.005). Psychological distress scores did not differ significantly between surgical history types (minor or major amputation or limb salvage, *p* = 0.655).

**Conclusions:** CMD in chronic wound patients is associated with higher psychological distress, greater pain, and lower function, with increased suicidal ideation. Routine psychiatric screening and integrating mental health services into multidisciplinary wound care could improve outcomes in this comorbid population.

## 68 165

### 68.1 Pain Intensity Impacts Functional and Psychological Outcomes in Limb Salvage Patients: The Need for Multidisciplinary Care

#### 68.1.1 Christopher Attinger, MD; Sami Ferdousian, MS; Ryan Lin, MD; Rachel Rohrich

68.1.1.1

**Background:** Microsurgical free tissue transfer (FTT) and partial foot amputation (PFA) are effective limb‐salvage options; however, pain intensity may affect patients’ societal functioning. This study analyzes patient‐reported outcome measures (PROM) in limb salvage patients with low and high pain intensity.

**Methods:** PROMs were collected via QR code for adult chronic lower extremity wound patients who underwent PFA and FTT, presenting to a tertiary wound center from June 2022 to October 2024. The Self‐Report Questionnaire (SRQ‐20), Connor‐Davidson Resilience Scale (CD‐RISC), and Lower Extremity Functional Scale (LEFS) were completed at 1, 3, and 6 months and 1, 2, and 5 years postoperatively.

**Results:** Of 106 survey sets, 63 were classified as lower pain and 43 as high pain. There was no significant difference in modified frailty index (1.88 ± 1 vs. 1.86 ± 1.3; *p* = 0.9) or Charlson Comorbidity index (4.9 ± 2.6 vs. 4.3 ± 2.6; *p* = 0.263) between low and high pain groups. The low pain group had higher mean overall function (LEFS; 51.1 ± 23.2 vs 33.7 ± 16.6; *p* = 0.001), resilience (CD‐RISC; 7.3 ± 1.3 vs. 6.4 ± 1.5; *p* = 0.001), and less psychological distress (SRQ‐20; 2.3 ± 2.9 vs. 4.8 ± 3.6; *p* = 0.001) compared to the high pain group.

**Conclusions:** Pain optimization is an essential part of optimizing a limb salvage patient's societal function. Limb salvage patients that experience high pain intensity may have better patient outcomes with a patient‐centered multidisciplinary care team.

## 69 166

### 69.1 Age Matters: Quality‐of‐Life Outcomes in Geriatric Patients Undergoing Below‐Knee Amputation

#### 69.1.1 Christopher Attinger, MD; Joshua Carreras; Sami Ferdousian, MS; Ryan Lin; Rachel Rohrich

69.1.1.1

**Background:** There is a lack of quality‐of‐life data of geriatric patients undergoing below the knee amputation (BKA). This study aims to analyze patient reported outcome measures (PROM) following below the knee amputations in patients below and above 70 years old.

**Methods:** PROMs were collected via QR code for adult chronic lower extremity wound patients who underwent BKA, presenting to a tertiary wound center from June 2022 to October 2024. The Self‐Report Questionnaire (SRQ‐20), PROM Information System Pain Intensity (PROMIS‐3a), Connor‐Davidson Resilience Scale (CD‐RISC), and Lower Extremity Functional Scale (LEFS) were completed at 1, 3, and 6 months and 1, 2, and 5 years postoperatively.

**Results:** Of 82 patients, 61 were < 70 years old and 21 were 70+ years old. Charlson Comorbidity Index was significantly higher in the 70+ years group compared to the < 70 years group (6.4 ± 2.1 vs. 3.9 ± 2.3; *p* = 0.001). The 70+ group had lower mean overall function (LEFS; 38.6 ± 22.4 vs 47.4 ± 24.8; *p* = 0.021) and lower pain intensity (PROMIS‐3a; 46.4 ± 10.6 vs. 50.8 ± 13.8; *p* = 0.029) compared to the < 70 group. There was no significant difference in mean overall resilience (CD‐RISC; 7 ± 1.4 vs. 6.9 ± 1.5; *p* = 0.66) or psychological distress (SRQ‐20; 2.6 ± 3.3 vs. 3.8 ± 4.4; *p* = 0.078) in the 70+ group versus the < 70 group.

**Conclusions:** 70+ year old BKA patients may require specialized patient‐oriented multidisciplinary care to optimize their postsurgical societal function.

## 70 167

### 70.1 Long Term Functional Outcomes Following Free Anterolateral Thigh Flap with Rolled Fascia Lata for Achilles Tendon Reconstruction

#### 70.1.1 Karen Evans, MD; Sami Ferdousian, MS; Ryan Lin, MD; Rachel Rohrich; Isabel Snee

70.1.1.1

**Background:** Achilles tendon injury with extensive soft tissue loss presents challenges to lower extremity (LE) reconstruction with functionally favorable results. The anterolateral thigh (ALT) flap with composite tissue using a rolled fascia lata (FL) is a reconstructive option in this population. This study evaluates the outcomes of microsurgical reconstruction for Achilles tendon and soft tissue defects.

**Methods:** A retrospective review of patients requiring LE free tissue transfer (FTT) between 2011 and 2023 was performed. Demographics, comorbid conditions, baseline functionality, reconstructive details, and wound characteristics were collected. Primary outcomes were flap success, return to functional ambulation, and complication rate.

**Results:** Twenty‐two patients underwent single‐stage FTT for both soft tissue coverage and Achilles tendon reconstruction. An ALT flap with attached FL extension rolled into a neo‐tendon was used in all 22 cases. The average age was 46.9 ± 16.1 years old, with a median Charlson Comorbidity Index of 0 (IQR:2). Average wound size was 77.0 ± 44.2 cm2. All patients (n=22, 100.0%) were ambulatory preoperatively. Flap success rate was 95.5% (n=21). Twelve patients (55%) had long term physical therapy follow up. At a median of 7.6 months (IQR:5.2), median ankle active range of motion (AROM) was 15 degrees (IQR:7) for dorsiflexion (DF) and 43.5 degrees (IQR:10) for plantarflexion (PF). Eleven (50%) patients had normal DF AROM (91.7%), and 9 (81.8%) patients had normal PF AROM. At a median of 2.3 months (IQR:2.8), all 22 patients (100.0%) returned to full weight bearing and ambulation. Median follow‐up time was 6.9 months (IQR:16.5).

**Conclusions:** The ALT free flap with an attached FL is a functional reconstructive technique for Achilles tendinous injury in the setting of large soft tissue defects. The composite free flap provides consistent operative success with robust patient return to ambulation and weight bearing. Further evaluation into patient reported outcomes can provide additional understanding of functionality.

## 71 168

### 71.1 Pain Intensity Differs, but Quality of Life Remains Comparable Across Limb‐Salvage and Below‐Knee Amputation in Chronic Wound Patients

#### 71.1.1 Christopher Attinger, MD; Danny Chamaa; Sami Ferdousian, MS; Ryan Lin, MD; Rachel Rohrich

71.1.1.1

**Background:** Choice of limb‐salvage approach depends on factors such as baseline function, wound extent, or wound complexity. The current study compares patient reported outcomes measures (PROMs) of patients who underwent limb salvage via mid‐foot amputation (MFA) or calcanectomy, to those that underwent BKA.

**Methods:** PROMs were collected via QR code for adult non‐healing lower extremity wound patients presenting to a tertiary wound center from June 2022 to May 2024. A cross‐sectional analysis of patients who underwent MFA, partial or total calcanectomy, or BKA was conducted. SRQ‐20, PROMIS‐3a, CD‐RISC, and LEFS were completed at up to 5 years postoperatively.

**Results:** Of 251 survey sets, 88 underwent MFA, 23 underwent calcanectomy, and 140 underwent BKA. Comorbidities were similar between groups. No significant differences were observed between MFA, calcanectomy, and BKA patients in mean overall function (LEFS; 43.4 ± 22.4 vs 40.6 ± 23.6 vs. 47.4 ± 25.1; *p* = 0.289), psychological distress ( SRQ‐20; 3.6 ± 3.7 vs 2.7 ± 2.6 vs. 3.1 ± 3.7; *p* = 0.436), resilience (CD‐RISC ; 6.8 ± 1.3 vs 7.4 ± 1.1 vs. 6.9 ± 1.6; *p* = 0.342), but significant difference was observed in mean overall pain intensity (PROMIS‐3a ;52.3 ± 11.6 vs. 41.5 ± 8.6 vs. 48.6 ± 13.2; *p* = 0.007).

**Conclusions:** The relative heterogeneity of PROMs across groups may reflect that limb salvage decisions were made for the intended patients. Chronically ill populations have similarly reported patient outcomes due to patient‐specific indications for surgery, multidisciplinary patient‐orientated decision making, and overall care.

## 72 169

### 72.1 The First Real‐World Multi‐Center Retrospective Outcomes following Transcatheter Arterialization of the Deep Veins for No‐Option Chronic Limb‐Threatening Ischemia

#### 72.1.1 Steven Abramowitz, MD; Nicole D’Ambrosio, MD; Kyle Reynolds, MD

72.1.1.1

**Background:** Up to 20% of patients with severe PAD and persisting ischemic ulcers or gangrene are ineligible for conventional endovascular or surgical revascularization options. These patients with no‐option chronic limb‐threatening ischemia (CLTI) are at high risk for major amputations and mortality. Transcatheter arterialization of the deep veins (TADV) has been shown as a viable treatment option in these patients with positive outcomes demonstrated in recent clinical studies. Here we report the real‐world outcomes in a retrospective single‐center study.

**Methods:** We performed a retrospective review of patients undergoing TADV procedures at our institution from April 2024 through February 2025. Technical success was defined as successful implantation of the TADV stent grafts. Clinical outcomes included amputation‐free survival, limb salvage, and survival rates.

**Results:** A total of 19 TADV patients were included in the analysis. The average age was 65 ± 12 years and 79% were male. Technical success was 100%. The median follow‐up was 166 days (range: 5‐320) post‐TADV. At the latest follow‐up, the limb salvage rate was 68% and the survival rate was 95%. All major amputations occurred within 6 months of the TADV procedure.

**Conclusions:** This real‐world retrospective study shows that TADV achieves high rates of limb salvage in no‐option CLTI patients who are at high risk for major amputation and other poor outcomes.

## 73 170

### 73.1 Resilience and Psychological Distress in Limb Salvage vs. Amputation: A 5‐Year Comparative Study

#### 73.1.1 Christopher Attinger, MD; Danny Chamaa; Sami Ferdousian, MS; Ryan Lin, MD; Rachel Rohrich

73.1.1.1

**Background:** Below knee amputations (BKA) may allow for a quicker return to ambulation however lower extremity (LE) free tissue transfer (FTT) often confers prolonged hospital recovery and rehabilitation. This study compares levels of resilience and psychological distress in comorbid patients with chronic LE wounds who underwent either BKA or limb salvage with FTT.

**Methods:** Using a cross‐sectional study design, we reviewed patients who underwent FTT or BKA and answered postoperative surveys from June 2022 to May 2024. Patient reported outcome measures included PROMIS‐3a, CD‐RISC, LEFS, and SRQ‐20, and were completed up to 5‐years postoperatively.

**Results:** A total of 213 surveys were completed by 82 patients; 50 underwent BKA and 32 underwent FTT. BKA patients reported overall less median pain than FTT patients (PROMIS‐T score; 43.1, IQR: 18.5 vs. 51.4, IQR: 28.6 *p* = 0.004). Median SRQ‐20 scores were < 10 at across all time periods for both BKA and FTT patients (no psychological distress). Both BKA and FTT patients demonstrated similar 3‐month (8, IQR: 2 vs. 8, IQR: 2, *p* = 0.81), 1‐year (8, IQR: 2 vs. 8, IQR: 1, *p* = 0.158), and 5‐year (6.7 ± 1.4 vs. 6.6 ± 1.3, *p* = 0.72) resilience (CD‐RISC). Across all time points, suicidal ideation was observed in 4 BKA patients (8%) compared to 0 FTT patients (p = 0.13).

**Conclusions:** Patients who undergo either FTT or BKA show comparably high resilience and no psychological distress up to 5 years postoperatively. Resiliency and psychological distress should continue to be assessed in patients who undergo lower extremity amputation and limb‐salvage procedures.

## 74 171

### 74.1 Overcoming Calcified Vessels in Limb Salvage: The Role of End‐to‐Side Technique with Saphenous Vein Interposition Grafts in Free Tissue Transfer

#### 74.1.1 Karen Evans, MD; Sami Ferdousian, MS; Nisha Gupta, MS; Ryan Lin, MD; Rachel Rohrich

74.1.1.1

**Background:** Atherosclerotic disease significantly increases complications in lower extremity (LE) free tissue transfer (FTT), particularly when donor and recipient vessels are calcified. This necessitates end‐to‐side (ETS) anastomosis to preserve distal perfusion, yet conventional techniques often fail due to loss of vascular compliance and size mismatch. The use of a saphenous vein interposition graft (SViG) has been previously described as a novel approach to address these challenges. This study aims to evaluate the short‐ and long‐term outcomes of SViG in patients with severe atherosclerosis undergoing LE FTT.

**Methods:** A single‐center retrospective review of 360 LE FTTs performed between 2011 and 2024 identified 40 patients (11.1%) requiring an SViG due to severe vessel calcification. Patient demographics, vascular characteristics, surgical details, and postoperative outcomes were analyzed. The primary outcomes of interest were flap success and limb salvage, with secondary outcomes including postoperative complications, time to ambulation, and long‐term follow‐up.

**Results:** The SViG cohort had an average age of 60.9 ± 11.5 years and a Charlson Comorbidity Index (CCI) of 5.2 ± 2.2, indicating high disease burden. Diabetes mellitus was the most common comorbidity (85.0%), followed by peripheral vascular disease (42.5%). Anterolateral thigh (ALT) flaps were the most common (47.5%), followed by vastus lateralis (32.5%). Fifteen patients (37.5%) required preoperative endovascular intervention. Short‐term complications included flap takeback in 10.0%, with an overall flap success rate of 95.0%. Median follow‐up was 14 months (IQR: 30.5) and 70.0% of patients were ambulatory postoperatively.

**Conclusions:** SViG provides a reliable flow‐sparing ETS technique in the setting of severe atherosclerosis, facilitating optimal pedicle arrangement and long‐term flap viability. Despite the high‐risk profile of this patient population, SViG demonstrated high success rates. These findings support the expanded use of SViG in lower extremity reconstruction for patients with severe vascular disease who may otherwise be unsuitable candidates for limb salvage.

## 75 172

### 75.1 Targeted Nerve Interventions in Highly Comorbid Through‐Knee Amputees: Advancing Palliative Care for Neuropathic Pain

#### 75.1.1 Ryan Bender, MD; Danny Chamaa; Sami Ferdousian, MS; Ryan Lin, MD; Rachel Rohrich

75.1.1.1

**Background:** Major lower extremity amputation (MLEA) is often necessary for patients with severe dysvascular conditions, extensive tissue loss, or non‐salvageable infections. Through‐knee amputation (TKA) is an underutilized MLEA technique that provides advantages in weight‐bearing and counterbalance for wheelchair mobility. However, post‐amputation neuropathic pain remains a significant barrier to recovery, with up to 85% of amputees experiencing phantom limb pain (PLP) or residual limb pain (RLP), leading to prolonged opioid dependence. Targeted muscle reinnervation (TMR) and regenerative peripheral nerve interface (RPNI) have emerged as promising strategies to mitigate neuropathic pain by providing physiologic targets for severed peripheral nerves. This study evaluates the impact of surgical nerve intervention on pain management and opioid dependence in TKA patients with severe disease burden.

**Methods:** A retrospective analysis of all TKA with peripheral nerve modification procedures performed at a single medical center between 2018 and 2024 was conducted. Inclusion criteria included patients who underwent TKA with primary TMR, RPNI, or end‐to‐end nerve coaptation. Patients with planned above‐knee conversion or inadequate follow‐up were excluded. Data collected included demographics, comorbidities, operative details, complications, pain scores, and medication usage at 1, 3, 6, and 12 months postoperatively. Trends in morphine milligram equivalents (MME) and neuroleptic (pregabalin) dosages were analyzed over 12 months.

**Results:** A total of 36 TKAs were performed in 32 patients with a mean age of 69.7 years. The most common indications for TKA were dysvascular ischemia (41.7%) and infection (38.9%). Comorbidities were prevalent, with 66.7% of patients having diabetes, 55.6% having peripheral vascular disease, and a mean charlson comorbidity index (CCI) of 6.6. TMR was performed in 72.2% of cases, while RPNI and end‐to‐end coaptation were performed in 13.9% each. The median postoperative hospital stay was 11 days. Complications were observed in 8.3% of patients, including stump infection (2.8%) and dehiscence (5.6%). Neuroma‐related revision surgery occurred in 5.7% of cases. Among patients initially ambulatory, 55.6% were fitted for a prosthesis, and 55.0% of those achieved ambulation, with a median time to prosthesis of 3.6 months and ambulation of 4.4 months. The overall mortality rate was 36.1%, with a median time to mortality of 7.2 months. Pain outcomes demonstrated a reduction in residual limb and phantom pain over time, with 63.9% of patients reporting no pain at final follow‐up. Narcotic consumption decreased over time, with 16.7% of patients using opioids at a mean follow‐up of 7.9 months, compared to 50.0% at 1‐month postoperatively. Mean MME also declined from 66.8 at 1‐month follow‐up to 45.0 at final follow‐up. Pregabalin use followed a similar trend, decreasing from a mean of 148.0 at baseline to 57.3 mg at final follow‐up.

**Conclusions:** TMR and RPNI demonstrate promise as palliative interventions for neuropathic pain in TKA patients with high disease burden. By addressing post‐amputation pain at its root cause, these techniques offer a non‐pharmacologic alternative to traditional pain management strategies. Future studies should further evaluate their role in optimizing palliative care pathways for this highly comorbid and medically complex patient population.

## 76 173

### 76.1 Elimination of Lag Time in Diabetic Wound Healing Using Intracellular ATP Delivery

#### 76.1.1 Mohammad Bayat, PhD; Sufan Chien, MD; Harshini Sarojini, PhD

76.1.1.1

**Background:** In the past century, extensive research and clinical explorations have focused on wound healing, but so far NO single treatment has shown significant diabetic wound healing. We have developed a new approach for wound care by intracellular ATP delivery using liposomes to encapsulate Mg‐ATP.

**Methods:** We used 25 rabbits, 15 were normal and 10 were one‐year diabetic. One ear was rendered ischemic and another ear was normal. Four circular full‐thickness wounds were created on the ventral side. ATp ‐vesicles and saline (control) were used daily.

**Results:** New tissue started to appear within 12‐24 h when ATp ‐vesicles were used. In the control wounds, no new tissue appeared until 5‐6 days. The average wound healing time in diabetic ischemic wounds was 19.55 days for ATp ‐vesicles vs. 29.10 days with saline (p = 0.0061).

A preliminary mechanistic study showed extremely early and rapid macrophage accumulation, in situ proliferation, M2 polarization, and direct collagen production to form extracellular matrix long before fibroblasts come into play when ATp ‐vesicles were used. If this can be duplicated in humans, it will be a breakthrough in medicine. Our studies have shown wounds treated by ATp ‐vesicles have higher expressions of macrophage markers, MCp ‐1, stem cell markers (CD44, CD106, CD146, and CD34) at day 1, and IL‐1ß and TNF‐a expressions and significantly higher VEGF‐A, VEGF‐D, and VEGFR‐2 expressions in early healing time when compared to the controls.

**Conclusions:** The extremely early expression of these factors leads to the rapid macrophage accumulation and wound healing. These results represent a new mechanism for enhanced diabetic wound healing.

## 77 174

### 77.1 Outcomes of a Two‐Stage Approach Using External Fixation to Internal Fixation in Charcot Neuroarthropathy: A Retrospective Case Series

#### 77.1.1 Claudia Barajas, DPM; Jennifer Buchanan, DPM, MS; Sharon Dei‐Tumi, DPM, MPH; Harry Schneider, DPM; Michael Theodoulou, DPM

77.1.1.1

**Background:** Charcot neuroarthropathy (CN) presents challenges in limb salvage, particularly in surgical stabilization. A two‐stage approach—external fixation (ex‐fix) followed by internal fixation (in‐fix)—has been proposed to optimize outcomes. This retrospective study evaluates clinical and radiographic results, analyzing complications, fusion rates, and amputation‐free survival across a cohort of CN patients who underwent this staged procedure.

**Methods:** This retrospective cohort study included 14 CN patients who underwent a two‐stage surgical approach. The cohort comprised 10 males and 4 females, with a mean age of 63 years (range: 53–79). Clinical data collected included demographics, hemoglobin A1c, Charcot location, surgical interventions, and post‐operative weight‐bearing timelines. Radiographic assessments were conducted to evaluate alignment and hardware stability. Follow‐up ranged from 10 to 46 months. The outcomes assessed were complications, weight‐bearing and amputation‐free survival.

**Results:** Full weight‐bearing occurred between 8 and 11 weeks, with some requiring extended offloading. Radiographs confirmed plantigrade alignment and stable hardware in most cases. Complications included post‐operative pain, pin site wounds (resolved after treatment), and prominent screw head penetration through the skin. One patient developed a septic ankle joint, leading to below‐knee amputation (BKA). Limb salvage was achieved in the remaining 13 patients, with no deep infections, hardware failures, or nonunions.

**Conclusions:** This staged approach provides structural stability and preserves soft tissue integrity. The successful limb salvage outcomes in most patients suggest its efficacy in CN management. However, the BKA case highlights the need for improved post‐operative monitoring and patient compliance. Future research should address patient‐specific risk factors.

## 78 175

### 78.1 A Clot to Handle: The Prevalence and Management of Preoperative Venous Thrombosis in Patients Undergoing Microsurgical Free Flap Reconstruction

#### 78.1.1 Cameron M. Akbari, MD; Christopher E. Attinger, MD; Kenneth L Fan, MD; Karen K. Evans, MD; Sami Ferdousian, MS; Leila I. Jones, BS; Karen R. Li, MD; Ryan P. Lin, MD; Rachel N. Rohrich, BS; Richard C. Youn, MD

78.1.1.1

**Background:** Venous pathology is an underappreciated yet critical factor in microsurgical limb salvage. While arterial inflow considerations dominate surgical planning, venous outflow is equally vital for flap survival. Patients requiring free tissue transfer (FTT) for lower extremity reconstruction frequently present with preoperative venous thrombosis (VT), yet there is little data guiding recipient vein selection and perioperative management in this high‐risk population.

**Methods:** A retrospective review of patients with preoperative VT undergoing lower extremity FTT at a single institution was conducted. VT characteristics (location, chronicity, occlusiveness) and venous reflux (VR) were assessed via preoperative venous duplex ultrasound. Microsurgical vein selection, perioperative anticoagulation strategies, and postoperative outcomes were analyzed.

**Results:** Among 279 FTT patients that underwent preoperative venous testing, 43 (15.4%) had preoperative VT, of which 34.9% had deep vein thrombosis (DVT), 60.5% had superficial venous thrombosis (SVT), and 4.7% had both. Venous reflux also was present in 77.5% of patients. There were two cases of takeback (4.7%) due to thrombosis, of which one flap was salvaged. By a median follow‐up duration of 9.7 months, a limb salvage rate of 88.4% was achieved.

**Conclusions:** Preoperative VT is common in microsurgical candidates for limb salvage and significantly influences management; however, it is not a contraindication to microsurgical limb salvage if proper adjustments are made perioperatively. Considerations in these patients include (1) routine use of venous ultrasound to identify VT; (2) perioperative anticoagulation management; and (3) selection of recipient veins that are unaffected by VT or reflux.

## 79 176

### 79.1 Exparel vs. Marcaine for Donor Site Analgesia in Atraumatic Lower Extremity Wound Split‐ Thickness Skin Grafting: Does Neuropathy Alter the Pain Equation?

#### 79.1.1 Sabrina DeLeonibus, MD, PhD, MS; Karen Evans, MD; Sami Ferdousian, MS; Ryan Lin; Rachel Rohrich

79.1.1.1

**Background:** Effective pain management at the donor site of split‐thickness skin grafts (STSG) is important, particularly in the challenging atraumatic lower extremity (LE) wound population. Exparel (liposomal bupivacaine) and Marcaine (bupivacaine) are commonly used for donor site analgesia, yet no studies have directly compared their efficacy in this specific patient population. Additionally, the role of neuropathy in postoperative pain perception in this context has not been well characterized. This study evaluates the efficacy of Exparel, Marcaine, and lidocaine in controlling donor site pain and also assesses differences in pain outcomes between neuropathic and non‐neuropathic patients.

**Methods:** A retrospective review was conducted on patients who underwent STSG for chronic atraumatic LE wounds at a single institution from December 2014 to December 2022. Patients received either Exparel, Marcaine, a combination of Exparel and Marcaine, or lidocaine for donor site analgesia. Primary outcomes included postoperative pain scores on postoperative day (POD) 0 and at the first follow‐up visit.

**Results:** A total of 246 patients were included, with a mean age of 62.8 ± 14.7 years. The distribution of analgesic strategies was as follows: Exparel (25.5%), Marcaine (21.6%), Exparel + Marcaine (12.0%), and lidocaine (40.9%). Median pain scores on POD 0 were significantly lower in the Exparel (0 [3]) and Marcaine (0 [2.5]) groups compared to lidocaine (2 [0], *p* = 0.020). At the first follow‐up, pain scores remained lowest in the Exparel (0 [4]) and Marcaine (0 [3]) groups compared to lidocaine (2.5 [5]), though this difference was not statistically significant (p = 0.241). When comparing neuropathic to non‐neuropathic patients, neuropathic patients experienced significantly lower postoperative pain both immediately after surgery (p = 0.008) and at the first follow‐up (p = 0.026). Among neuropathic patients, pain scores did not significantly differ between analgesic groups at any time point.

**Conclusions:** This study is the first to compare Exparel and Marcaine for donor site analgesia in patients undergoing STSG for chronic atraumatic LE wounds. Both agents provided superior immediate postoperative pain control compared to lidocaine, with Exparel demonstrating a potential trend toward prolonged analgesia. Additionally, neuropathic patients exhibited significantly less postoperative pain than their non‐neuropathic counterparts, independent of the analgesic used. These findings highlight the importance of individualized pain management strategies in this population.

## 80 177

### 80.1 Evaluating Distal Tibial Distraction Osteogenesis for Limb Salvage in Charcot Neuroarthropathy: A Promising Surgical Approach

#### 80.1.1 Jayson Atves, DPM; Tiffanie Liu, DPM; Ali Qadri, DPM; Ali Rahnama, DPM; Isabel Snee

80.1.1.1

**Background:** Charcot neuroarthropathy of the hindfoot and ankle is a debilitating condition that causes severe bone and joint destruction, leading to deformities, chronic infections, and gait issues. Traditional surgical management, such as hindfoot arthrodesis, often faces high complication and non‐union rates due to poor vascularity and disorganized bone structure. Recently, distal tibial distraction osteogenesis has emerged as a promising alternative to enhance vascularity and improve fusion outcomes. This study aims to evaluate the effectiveness of this combined approach in patients with Charcot neuroarthropathy by assessing complication rates, union rates, and overall limb salvage, offering new insights into advanced management strategies for this complex condition.

**Methods:** We performed a comparative retrospective analysis of 8 patients who were evaluated at Georgetown University Hospital between 2019 to 2023. Outcomes were defined as success of limb salvage, whether the patient was able to fully weightbear, complications which included amputation rates, functional measures and infection rates.

**Results:** The study included 8 patients with a follow‐up range of 120 to 1333 days and a mean follow‐up duration of 518 days. On average, patients spent 85.13 days in external fixation and took 155.75 days to ambulate. The salvage rate at six months was 100%, with 71.40% ambulating at baseline. Mean HgbA1c was 7.56, and mean BMI was 32.1. Readmission rates were 25% for both initial and final procedures, and the revision rate was 62.50%. The mean age was 59.88 years, and the mean Charlson Comorbidity Index was 4. Additionally, 25% of patients had a history of ipsilateral amputations, reflecting the complexity of their medical backgrounds.

**Conclusions:** This study highlights the potential of a combined surgical approach involving distal tibial distraction osteogenesis and tibiotalocalcaneal arthrodesis in patients with Charcot neuroarthropathy of the hindfoot and ankle. With an encouraging salvage rate of 100% a substantial proportion of patients were able to ambulate following treatment. The findings suggest this technique may effectively address the complexities associated with this debilitating and surgically challenging condition. Despite challenges such as revision rates and a significant percentage of patients with previous amputations, the promising outcomes warrant further investigation into the long‐term efficacy and safety of this approach. A collaborative, multidisciplinary approach that encompasses careful patient selection, optimization of comorbidities, and ongoing postoperative management will be essential to fully realize the benefits of this innovative surgical strategy.

## 81 178

### 81.1 The Effect of Major Unplanned Reoperations on Patient Reported Outcomes in Patients Undergoing Charcot Reconstruction

#### 81.1.1 Christopher Berkelbach, DPM; Madiera Curry, DPM; Hiba Mohiuddin, DPM; Jacob Wynes, DPM

81.1.1.1

**Background:** Patients with Charcot neuroarthropathy have a lifetime risk of amputation of 15%. Improvements in the surgical management of Charcot have increased salvageability, however patients often endure a tumultuous journey to achieve limb preservation, including oftentimes numerous unplanned surgeries, which takes an economic and emotional toll. We evaluated the impact the number of unplanned major reoperations may have on patient reported outcomes in patients undergoing Charcot reconstruction surgery.

**Methods:** We performed a retrospective review of 145 patients treated surgically for Charcot neuroarthropathy. Patients with less than 12 months of post‐operative follow‐up were excluded. All operative interventions on the affected limb were categorized as major or minor, planned or unplanned, and to have taken place before or after the index procedure. Patient reported outcomes were collected using five LIMB‐Q scales: Physical Function, Life Impact, Psychological, Information, and Decision.

**Results:** Following the index Charcot reconstruction procedure, 72% of patients underwent 1 or more unplanned reoperations on the affected limb (range 1‐11). The mean number of unplanned reoperations per limb was 2.4. 43% of patients underwent 1 or more major unplanned reoperations. Patients who underwent 3 major unplanned reoperations had the lowest LIMB‐Q scores for all 5 scales collected. Patients undergoing 4 or more major unplanned reoperations had comparable LIMB‐Q scores to patients who underwent zero, one or two major unplanned reoperations.

**Conclusions:** Patients undergoing Charcot reconstruction should be informed of the high likelihood of requiring unplanned reoperation. The number and type of major unplanned reoperations, however, may not affect the overall outcome.

## 82 179

### 82.1 Effect of Travel Distance and Time on Clinical Outcomes in Patients Undergoing Charcot Reconstruction

#### 82.1.1 Christopher Berkelbach, DPM; Jacob Wynes, DPM

82.1.1.1

**Background:** There is a high morbidity and mortality related to Charcot neuroarthropathy and its surgical and non‐surgical management. A recent study showed good outcomes for Charcot patients with early referral to a tertiary care center. We investigated the effect of travel distance and time on clinical outcomes in patients undergoing treatment for Charcot neuroarthropathy at a large urban tertiary care specialty clinic in Maryland.

**Methods:** We performed a retrospective review of 145 patients treated surgically for Charcot neuroarthropathy. Patients with less than 12 months of post‐operative follow‐up were excluded. We used the directions feature on Google Maps to estimate the distance and driving time from the patient's home address to the outpatient treatment facility and hospital.

**Results:** The average distance and driving time to the main outpatient treatment facility was 35.2 miles and 40.8 min, respectively. Patients who lived greater than 50 miles from the outpatient treatment facility underwent an average of 4.5 unplanned reoperations compared with 2.3 unplanned reoperations for those who lived less than 50 miles from the outpatient treatment facility. Patients who lived greater than 50 miles from the outpatient treatment facility were more likely to have complications with their external fixator including pin site irritation and pin breakage/loosening.

**Conclusions:** Patients with Charcot neuroarthropathy traveling a great distance for treatment at a tertiary care specialty clinic may be at increased risk of complications and re‐operation following surgical management.

## 83 180

### 83.1 HOYA Foot Care Clinic: Expanding Access and Education in Diabetic Limb Salvage

#### 83.1.1 John DiBello, MS; Abigail Escobar; Izzy Jones

83.1.1.1

**Background:** Mobile free clinics are critical for early prevention, screening and management of diabetic foot complications among populations at high risk for limb loss. HOYA Foot Care Clinic (HFCC) is a student‐run mobile initiative that provides foot exams, hygienic treatment, and wound care to underserved communities in Washington D.C. HFCC augments medical education by exposing students to podiatric medicine in underserved settings while embodying Georgetown's commitment to Cura Personalis and People for Others.

**Methods:** HFCC partners with local homeless shelters Medical students conduct comprehensive foot exams and identify diabetic foot complications, including neuropathy, venous insufficiency, and infection. Patients without an established healthcare network are referred to Unity Health Care or MedStar Podiatry for ongoing management of diabetes and other chronic conditions.

**Results:** Between August 3, 2023 and February 27, 2025, HFCC held 15 mobile clinics in collaboration with six community organizations across Washington, D.C., providing comprehensive foot care to 280 patients. Of those evaluated, 70 individuals received referrals to Unity Health Care or MedStar Podiatry for ongoing management of acute and chronic needs.

**Conclusions:** HFCC exemplifies how student‐run mobile clinics can sustainably collaborate with community organizations to address immediate foot care needs and support long‐term diabetic management. This model of care expands access to ongoing health services for underserved populations, reducing the risk of life‐limiting complications and limb loss. Additionally, HFCC enhances medical education by exposing students to complex systemic inequities, increases visibility of podiatric medicine, and furthers Jesuit, Catholic mission of Georgetown University through community outreach and continued care.

## 84 181

### 84.1 Innovative Strategies for Complex Ankle Conditions with Custom PEEK Implants; A Tale of an Ankle with Charcot Neuroarthropathy

#### 84.1.1 Paul Cooper, MD; Tiffanie Liu, DPM; Ali Qadri, DPM

84.1.1.1

**Background:** Traditionally, ankle fusion in cases of significant bone loss such as in the case of Charcot Neuroarthropathy has employed a range of techniques and materials. Allografts and autografts are frequently utilized to address bone defects. These allografts, while effective, come with a higher risk of complications, including immune rejection, donor site morbidity, and lengthy recovery times. The emergence of metallic cages as a replacement to femoral head in ankle fusion can provide added stability, but they are not without their own challenges, such as stress shielding, potential failure under excessive load, and limitations in screw placement due to the inability to secure screws through the cage, which can hinder proper stabilization and healing.

In recent years, advancements in implant technology have introduced custom polyetheretherketone (PEEK) implants as a promising alternative for improving outcomes in ankle fusion surgeries. Unlike allografts and autografts, PEEK implants are biocompatible, reducing the risk of adverse reactions. Moreover, they can be filled with autologous bone, eliminating the need for additional surgical sites and promoting better integration with the surrounding tissue. The material has high tensile strength and an elastic modulus that closely matches that of bone, allowing for better load distribution and reduced risk of implant failure.

This case study showcases the use of a custom PEEK implant in a case due to bone loss secondary to Charcot Neuroarthropathy.

**Methods:** We performed a retrospective analysis of one patient who was evaluated at Georgetown University Hospital between 2022 to 2024. Outcomes were defined as success of limb salvage and fusion at the ankle.

**Results:** 61‐year‐old male presented with a left ankle wound complicated by a MRSA infection, leading to osteomyelitis of the distal tibia and fibula. The patient also had Charcot arthropathy. He underwent irrigation and debridement (I&D) with extensive resection of the osteomyelitic tissue in the distal tibia and remaining talus, followed by the application of a multiplanar external fixator. Four days later, a repeat I&D was performed due to further osteomyelitis of the calcaneus and tibia, leading to the insertion of an antibiotic spacer and adjustment of the external fixator. Two months later, the external fixator was removed, and the patient underwent pantalar arthrodesis using a custom PEEK implant and a retrograde intramedullary nail to stabilize the fusion.

**Conclusions:** This case illustrates the complexities of managing Ankle Charcot Neuroarthropathy with active infection in an elderly patient through multiple surgical interventions. Infection management became crucial and included multiple I&D's, an antibiotic spacer with exchange and application of an external fixator.

The final outcome, featuring the successful implantation of a custom PEEK implant with an intramedullary nail, underscores the advantages of PEEK over traditional allograft femoral head options. PEEK offers superior biocompatibility, mechanical strength, and a reduced risk of complications, such as graft rejection or failure, which are common with allografts. Achieving fusion at one year highlights the potential of PEEK technology in improving outcomes for complex cases and positions it as a promising alternative to allograft procedures in ankle fusions.

Additionally, the use of PEEK mitigates challenges typically seen with metallic cages, such as stress shielding and difficulties in securing screws effectively. This enhancement in stability and fusion success is key in promoting optimal healing. However, the high cost of PEEK implants remains a significant limitation, making careful patient selection crucial for success. Despite this, the material's lightweight nature, resistance to fatigue, and favorable mechanical properties offer significant improvements in patient outcomes.

The successful fusion achieved at one year post‐surgery reflects the importance of personalized treatment strategies and ongoing research to refine surgical techniques and implant materials. Further studies are necessary to assess the long‐term safety and effectiveness of PEEK implants in orthopedic applications and to better understand their role in improving outcomes for complex ankle pathologies in older patients.

## 85 182

### 85.1 Intraoperative Vasopressor Use Does Not Adversely Impact Free Flap Outcome in Lower Extremity Limb Salvage Procedures

#### 85.1.1 Karen Evans, MD; Sami Ferdousian, MS; Ryan Lin, MD; Rachel Rohrich; Julie Suh

85.1.1.1

**Background:** The use of vasopressors (VP) during microsurgical reconstruction is debated. Their effect in the comorbid lower extremity (LE) wound population remains unstudied. This study characterizes the impact of intraoperative VP use in LE free tissue transfer (FTT) for limb salvage.

**Methods:** A review of LE FTT from February 2017 to June 2024 was conducted.

**Results:** Of 259 LE FTT were performed, VP was used in 180 cases (69.5%). Most VP were administered via intermittent bolus only (76.1%) or combined with continuous infusion (23.3%). ASA Class was significantly higher in the VP group compared to controls (p = 0.001). The VP group trended to have higher median Charlson Comorbidity Indices (4, IQR: 3 vs. 3, IQR: 3; *p* = 0.057), and rates of diabetes (65.0% vs. 53.2%, *p* = 0.072), peripheral vascular disease (63.3% vs. 51.9%, *p* = 0.084), and chronic kidney disease (14.4% vs. 6.3%, *p* = 0.094). Rate of flap takeback, thrombosis, or flap success did not differ between groups. By a median long‐term follow up of 24.5 (IQR: 39.2) months, rates of major limb amputation (VP: 10.6% vs. control: 7.5%, *p* = 0.458) and mortality (4.7% vs. 2.7%, *p* = 0.353) were similar between groups. Univariate analysis demonstrated VP use significantly increased the odds of any flap complication, however this did not remain significant on multivariate analysis (OR: 1.1, CI: (0.2, 5.1), *p* = 0.892).

**Conclusions:** In this complex population, intraoperative use of VP does not independently impact flap viability or limb salvage. These data may provide reassurance for the safe use of VP during microsurgical reconstruction in comorbid populations.

## 86 183

### 86.1 Galangin's Impact on Expedite Wound Healing in Diabetic Rat Models

#### 86.1.1 Fahad Al‐Abbasi, PhD; Ashraf Abdel‐Naim, PharmD; Khadijah Alkinani, PhD

86.1.1.1

**Background:** Diabetes poses a global health concern, impacting millions worldwide. A critical complication of diabetes is impaired wound healing, especially in the form of diabetic foot ulcers (DFUs), often culminating in lower limb amputation.

This study centers on Galangin (GAL), a flavonoid with anti‐inflammatory properties. The objective is to explore the potential effects of GAL in accelerating wound healing process in diabetic rat models.

**Methods:** Diabetes was induced in rats using Streptozocin, followed by creating wounds. Rats were divided into treated and control groups. Wound healing progression was assessed through observation and analysis. Specifically, GAL's potential to influence wound closure, oxidative status, and inflammatory markers was evaluated. Histopathological analysis was performed using hematoxylin and eosin (H&E) or Masson's trichrome staining techniques. Additionally, quantitative real‐time PCR (qRT‐PCR) was conducted on excised tissues to assess the expression of collagen type I alpha 1 (Col1A1). Immunohistochemical analysis of vascular endothelial growth factor A (VEGF‐A) and transforming growth factor beta 1 (TGF‐ß1) were also carried out to shed some light on the mechanism of wound healing.

**Results:** GAL treatment significantly reduced oxidative stress by decreasing malondialdehyde (MDA) and increasing superoxide dismutase (SOD) activity. It also decreased pro‐inflammatory cytokines interleukin‐6 (IL‐6) and tumor necrosis factor alpha (TNF‐a). Additionally, GAL increased VEGF‐A, and TGF‐ß1 protein expression. Histopathological analysis revealed enhanced collagen deposition, keratinization, and epidermal proliferation. Furthermore, GAL treatment significantly upregulated mRNA expression of Col1A1, supporting its role in tissue repair and angiogenesis.

**Conclusions:** In conclusion, GAL represents a promising therapies as a topically applied to improve wound healing.

## Conflicts of Interest

The authors declare no conflicts of interest.

## Data Availability

Data is available upon request from the authors.

